# Supplementary material for: Characterizing the interactions between classical and community-aware centrality measures in complex networks
Source: Sci Rep. 2021 May 12;11:10088. doi: 10.1038/s41598-021-89549-x (PMC8115665; doi:10.1038/s41598-021-89549-x)
Supplement: Supplementary file 1 — Supplementary Information. [file 41598_2021_89549_MOESM1_ESM.pdf]

Supplementary Information for Characterizing the Interactions Between Classical and Community-aware Centrality Measures in Complex Networks

Stephany Rajeh[1,*], Marinette Savonnet[1], Eric Leclercq[1], and Hocine Cherifi[1]

[1]LIB EA 7534, University of Burgundy, Dijon, France
[*]stephany.rajeh@u-bourgogne.fr

ABSTRACT

Identifying vital nodes in networks exhibiting a community structure is a fundamental issue. Indeed, community structure is one of the main properties of real-world networks. Recent works have shown that community-aware centrality measures compare favorably with classical measures agnostic about this ubiquitous property. Nonetheless, there is no clear consensus about how they relate and in which situation it is better to use a classical or a community-aware centrality measure. To this end, in this paper, we perform an extensive investigation to get a better understanding of the relationship between classical and community-aware centrality measures reported in the literature. Experiments use artificial networks with controlled community structure properties and a large sample of real-world networks originating from various domains. Results indicate that the stronger the community structure, the more appropriate the community-aware centrality measures. Furthermore, variations of the degree and community size distribution parameters do not affect the results. Finally, network transitivity and community structure strength are the most significant drivers controlling the interactions between classical and community-aware centrality measures.

Supplementary Figures

Heatmaps of Synthetic Networks

- Supplementary Fig S1 shows the heatmaps of Kendall's Tau correlation between classical (α_i) and community-aware (β_j) centrality measures of LFR synthetic networks generated at a fixed degree distribution exponent ($\gamma = 2.7$), fixed community size distribution ($\theta = 2.7$), and an increasing mixing parameter ($\mu = 0.10$ to $\mu = 0.40$).

- Supplementary Fig S2 shows the heatmaps of Kendall's Tau correlation between classical (α_i) and community-aware (β_j) centrality measures of LFR synthetic networks generated at a varying degree distribution exponent ($\gamma = [2, 2.7, 3]$), fixed community size distribution ($\theta = 2.7$), at a weak community structure ($\mu = 0.70$).

- Supplementary Fig S3 shows the heatmaps of Kendall's Tau correlation between classical (α_i) and community-aware (β_j) centrality measures of LFR synthetic networks generated at fixed degree distribution exponent ($\gamma = 2.7$), varying community size distribution ($\theta = [2, 2.7, 3]$), at a weak community structure ($\mu = 0.70$).

- Supplementary Fig S4 shows the histograms of Kendall's Tau correlation between classical (α_i) and community-aware (β_j) centrality measures divided into local, global, and mixed groups. Networks have their respective degree distribution exponent varied ($\gamma = [2,3]$) at a strong community structure ($\mu = 0.05$).

- Supplementary Fig S5 shows the histograms of Kendall's Tau correlation between classical (α_i) and community-aware (β_j) centrality measures divided into local, global, and mixed groups. Networks have their respective degree distribution exponent varied ($\gamma = [2,3]$) at a weak community structure ($\mu = 0.70$).

- Supplementary Fig S6 shows the histograms of Kendall's Tau correlation between classical (α_i) and community-aware (β_j) centrality measures divided into local, global, and mixed groups. Networks have their respective community size distribution exponent varied ($\theta = [2,3]$) at a strong community structure ($\mu = 0.05$).

- Supplementary Fig S7 shows the histograms of Kendall's Tau correlation between classical (α_i) and community-aware (β_j) centrality measures divided into local, global, and mixed groups. Networks have their respective community size distribution exponent varied ($\theta = [2,3]$) at a weak community structure ($\mu = 0.70$).

Heatmaps of Real-World Networks

- Supplementary Figs S8, S9, S10, S11, S12, S13, S14, and S15 show the heatmaps of Kendall's Tau correlation between classical (α_i) and community-aware (β_j) centrality measures of real-world networks having their community structure identified using the Infomap community detection algorithm.
- Supplementary Figs S16, S17, S18, S19, S20, S21, S22, S23, and S24 show the heatmaps of Kendall's Tau correlation between classical (α_i) and community-aware (β_j) centrality measures of real-world networks having their community structure identified using the Louvain community detection algorithm.

Supplementary Tables

- Supplementary Table 1 and Table 2 report the macroscopic and mesoscopic characteristics of the networks, respectively.
- Supplementary Table 3 and Table 4 report the simple linear regression estimates using ordinary least squares and weighted least squares, respectively.

Supplementary Note 1: Classical and Community-aware Centrality Measures

Classical Centrality Measures

Different classical centrality measures are used in order to inspect their relationship with community-aware centrality measures. Consider the function $\alpha(i)$ as a classical centrality measure. They are based on viewing the network from a local and global perspective. In this paper, ten classical centrality measures are used. For more information about the implementation and other centrality measures, one can refer to[1].

- Let $G(V,E)$ be an undirected and unweighted graph where V is the set of nodes and $E \subseteq V \times V$ is the set of edges and $N = |V|$ is the total size of the network.
- Let the adjacency matrix $A = (a_{i,j})$ describe the connectivity of the graph G such that $a_{i,j} = 1$, if node i is connected to node j and $a_{i,j} = 0$, otherwise.
- Let the neighborhood of any node i be defined as the set $\mathcal{N}_p(i) = \{j \in V : (i,j) \in E\}$ at length p, where $p = 1,2,...,D$. D is the diameter of G. Accordingly, two nodes are neighbors of order A^p if there's a minimal path connecting them at p steps.

Local Measures

Local measures consider only the node's neighborhood to calculate its centrality.

Degree centrality. Degree centrality is based on the direct connections a node has. It can be defined as:

$\alpha_d(i) = \sum_{j=1}^{N} a_{ij}$

where a_{ij} is obtained from A^1, 1-step neighborhood (p=1).

Leverage centrality. Leverage centrality, unlike other measures, is signed. If a node has less connections compared to its direct neighbors, it has a negative centrality. In contrast, if it has more connections than its neighbors, it will obtain a positive centrality. It can be defined as:

$\alpha_{lev}(i) = \frac{1}{k_i} \sum_{j=1}^{N} \frac{k_i - k_j}{k_i + k_j}$

where k_i and k_j are the degrees of nodes i and j, respectively.

Laplacian centrality. Laplacian centrality is based on the idea that removing an important node would result in damaging the network. It is based on the Laplacian energy and can be defined as:

$\alpha_{lap}(i) = k_i^2 + k_i + 2\sum_{j \in \mathcal{N}_1(i)} k_j$

where k_i and k_j are the degrees of nodes i and j, respectively, and $\mathcal{N}_1(i)$ is the set of direct neighbors of node i.

Diffusion Degree centrality. Diffusion degree centrality is based on the cumulative diffusive influence of the node itself and its neighbors. It is defined as:

$\alpha_{dif}(i) = \varpi_i \times \alpha_d(i) + \sum_{j \in \mathcal{N}_1(i)} \varpi_j \times \alpha_d(j)$

where k_i and k_j are the degrees of nodes i and j, respectively, ϖ_i and ϖ_j are the propagation probabilities of nodes i and nodes j, respectively, and $\mathcal{N}_1(i)$ is the set of direct neighbors of node i. Propagation probability ϖ is set to 1 for all nodes.

Maximum Neighborhood Component centrality (MNC). Maximum Neighborhood Component centrality quantifies a node's influence based on the size of the largest connected component that can be attained from its direct neighborhood. It is defined as:

$\alpha_m(i) = |LCC \in \mathcal{N}_1(i)|$

where $\mathcal{N}_1(i)$ is the set of direct neighbors of node i.

Global Measures

Global measures consider the overall network topology to compute the centrality of a node.

Betweenness centrality. Betweenness centrality is based on how many times a node is situated in the shortest path between other two nodes. It is defined as:

$\alpha_b(i) = \sum_{s,t \neq i} \frac{\sigma_i(s,t)}{\sigma(s,t)}$

where $\sigma(s,t)$ is the number of shortest paths between nodes s and t and $\sigma_i(s,t)$ is the number of shortest paths between nodes s and t that pass through node i.

Closeness centrality. Closeness centrality is based on how close on average a node is to all other nodes in the network. It is defined as:

$\alpha_c(i) = \frac{N-1}{\sum_{j=1}^{N-1} d(i,j)}$

where $d(i,j)$ is the shortest path distance between node i and j.

Katz centrality. Katz centrality quantifies a node's importance based on the influence of all the other nodes on it and their subsequent distances. As the distance of a node increases, its influence diminishes. It is defined as:

$\alpha_k(i) = \sum_{p=1} \sum_{j=1} s^p a_{ij}^p$

where a_{ij}^p is the connectivity of node i with respect to all the other nodes at A^p and s^p is the attenuation factor where $s \in$ [0,1].

PageRank centrality. PageRank centrality quantifies a node's importance based on the influence of all other nodes on it as a Markov chain process. The undirected version is defined as:

$\alpha_p(i) = \frac{1-d}{N} + d \sum_{j \in \mathcal{N}_1(i)} \frac{\alpha_p(j)}{k_j}$

where $\alpha_p(i)$ and $\alpha_p(j)$ are the PageRank centralities of node i and node j, respectively, $\mathcal{N}_1(i)$ is the set of direct neighbors of node i, k_j is the number of links from node j to node i, and d is the damping parameter where $d \in$ [0,1]. The damping parameter d is set to 0.85.

Subgraph centrality. Subgraph centrality quantifies a node's importance based on its participation in closed walks (each corresponding to a subgraph), starting and ending with the same node. Additionally, smaller subgraphs are given more weight. It can be calculated using the spectral decomposition of the adjacency matrix A:

$\alpha_s(i) = \sum_{j=1}^{N} (v_j^i)^2 e^{\lambda_j}$

where v_j refers to an eigenvector of the adjacency matrix A, associated with its eigenvalue λ_j.

Community-aware Centrality Measures

There are nine community-aware centrality measures used in this study. One of them, called modular centrality[2], extends the classical centrality measures by incorporating information from the intra-community links and inter-community links, separately. Hence, a total of 28 community-aware centrality measures are present. Note that in this paper, non-overlapping networks are used (i.e. each node belongs to a single community). Consider the function $\beta(i)$ as a community-aware centrality measure.

Measures based on intra-community links

Local community-aware centrality measures quantify the local influence of a node in its community based on its intra-community links and discard inter-community links.

Modular centrality based on local component. Modular centrality based on local component[2] of a network G_l only takes into consideration intra-community links of nodes. After obtaining the community structure C, a set of communities $C = \{c_1, c_2, ..., c_k\}$, G_l is obtained by the union of all disjoint communities $\bigcup_{k=1}^{N_c} c_k$ where $N_c = |C|$ is the total number of communities and c_k is the k-th isolated community. Each component in G_l is obtained by removing the inter-community links.

All classical centrality measures $\alpha(i)$ can be computed based on the local component G_l. We denote $\beta^L(i)$ as any selected classical centrality measure calculated on node $i \in G_l$.

Measures based on inter-community links

Global community-aware centrality measures quantify the global influence of a node through its external connections outside its community (inter-community links) or through its significance in the overall network by acting as a bridge between communities.

Modular centrality based on the global component. Modular centrality based on global component[2] of a network G_g only takes into consideration inter-community links of nodes. The process is similar to the process of obtaining the local component (G_l), however in this case all intra-community links are removed. $G_g = \bigcup_{r=1}^{p} s_p$ where $S = \{s_1, s_2, ..., s_p\}$ is the set of connected components after the removal of intra-community links and $p = |S|$ is the size of set S.

All classical centrality measures $\alpha(i)$ can be computed based on the global component G_g. We denote $\beta^G(i)$ as any selected classical centrality measure calculated on node $i \in G_g$.

Number of Neighboring Communities centrality (NNC). Number of Neighboring Communities centrality[3] of a node is based on the number of communities it can reach in one hop. As simulations indicate, it is well suited for networks with medium community strength (i.e. intra-community links equal inter-community links in the network). For a node in community $c_k \subset C$, NNC is defined as follows:

$\beta_{NNC}(i) = \sum_{c_l \subset C \setminus c_k}^{N} \bigvee_{j \in c_l} a_{ij}$

where $\bigvee_{j \in c_l} a_{ij} = 1$ when node i is connected to at least one node j in community c_l.

Bridging centrality (BC). Bridging centrality[4] combines betweenness centrality and bridging coefficient to identify bridging nodes. The bridging coefficient is based on the node's proximity to high degree nodes. Bridging centrality of a node is defined as:

$\beta_{BC}(i) = \alpha_b(i) \times \mathbb{B}(i)$

where $\alpha_b(i)$ is the classical betweenness centrality of node i and $\mathbb{B}(i) = \frac{k_i^{-1}}{\sum_{j \in \mathcal{N}_1(i)} k_j^{-1}}$ is the bridging coefficient where $\mathcal{N}_1(i)$ is the set of direct neighbors of node i.

Measures based on intra-community and inter-community links

Mixed community-aware centrality measures consider the node's local and global influence concurrently through its intra-community and inter-community links.

Comm centrality. Comm centrality[5] accounts for the presence of a hub role and a bridge role of a node, simultaneously. It combines the intra-community and inter-community links in a non-linear manner. To give more weight to bridges, since they are rare compared to hubs, the total inter-community links of a node is raised to a power of 2. It is defined as follows:

$$\beta_{Comm}(i) = (1+\mu_{c_k}) \times \left(\frac{k_i^{intra}}{max_{(j \in c)} k_j^{intra}} \times R\right) + (1-\mu_{c_k}) \times \left(\frac{k_i^{inter}}{max_{(j \in c)} k_j^{inter}} \times R\right)^2$$

where μ_{c_k} is the fraction of inter-community links over the total community links in community c_k, and R is a constant to scale intra-community and inter-community values to the same range.

Community-based Mediator centrality (CBM). Community-based Mediator centrality[6] takes into consideration the intra-community and inter-community link density of a node. It is based on the entropy of the given densities. The strategy aims to select the nodes that act as intermediaries for receiving and spreading information to the community the node belongs to and to different communities. It is defined as follows:

$\beta_{CBM}(i) = H_i \times \frac{k_i^{tot}}{\sum_{i=1}^{N} k_i^{tot}}$

where $H_i = [-\sum \rho_i^{intra} log(\rho_i^{intra})] + [-\sum \rho_i^{inter} log(\rho_i^{inter})]$ is the entropy of node i based on its ρ^{intra} and ρ^{inter} which represent the density of the communities a node links to (either its community or external communities), k_i^{tot} is the total degree of node i, and $\sum_{i=1}^{N} k_{tot\, i}$ is the total degrees in the network.

Community Hub-Bridge centrality (CHB). Community Hub-Bridge centrality[3], similar to Comm centrality, accounts for the presence of a hub role and a bridge role of a node. It combines the intra-community and inter-community links by weighting the former with the community size and the latter with the number of neighboring communities. For a node in community $c_k \subset C$, CHB is defined as follows:

$\beta_{CHB}(i) = h_i(c_k) + b_i(c_k)$

where the hub influence is given by $h_i(c_k) = |c_k| \times k_i^{intra}$ and the bridge influence is given by $b_i(c_k) = \beta_{NNC}(i) \times k_i^{inter}$. Indeed, CHB uses NNC in its formulation.

Community-based centrality (CBC). Community-based centrality[7] extends degree centrality taking into consideration the type of links (inter/intra) scaled with the size of their successive communities. It is defined as follows:

$\beta_{CBC}(i) = \sum_{c=1}^{N_c} k_{i,c} \left(\frac{n_c}{N}\right)$

where N_c is the total number of communities, $k_{i,c}$ is the number of links node i has in a given community c (can be inter-community or intra-community links), n_c is the number of nodes in community c, and N is the total size of the network.

Participation Coefficient (PC). Participation coefficient[8] of a node takes into consideration the distribution of its links inside and outside its community. If a node has all its links inside the community it belongs to, it will obtain a value of 0. While if a node has its links uniformly distributed across all communities, it will obtain a value of 1. Participation coefficient is defined as follows:

$\beta_{PC}(i) = 1 - \sum_{c=1}^{N_c} \left(\frac{k_{i,c}}{k_i^{tot}}\right)^2$

where N_c is the total number of communities, $k_{i,c}$ is the number of links node i has in a given community c (can be inter-community or intra-community links), and k_i^{tot} is the total degree of node i.

K-shell with Community centrality. K-shell with Community centrality[9] of a node applies k-shell hierarchical decomposition while taking into consideration the community structure. First, k-shell is applied separately on each of the local component and global component. Then, both values are weighted by a parameter δ to preferentially select either hubs or nodes. It is defined as follows:

$\beta_{ks}(i) = \delta \times \beta_{ks}^{L}(i) + (1-\delta) \times \beta_{ks}^{G}(i)$

where δ is set to be a value between [0,1]. A value of 1 means hub nodes are preferred, while a value of 0 means bridge nodes are. A value of 0.5 is set for δ in this study.

Supplementary Note 2: Macroscopic and Mesoscopic Topological Properties

Macroscopic Topological Properties

Macroscopic characteristics of a network characterize the whole network. The macroscopic characteristics used in this study, totaling seven, are the main ones used in literature[10].

Density. Density is used to quantify the number of edges over all possible edges that can be obtained in a network. It is defined as:

$\nu = \frac{2|E|}{N(N-1)}$

where $|E|$ is the total number of edges and N is the total number of nodes.

Transitivity. Transitivity measures the fraction of triangles in a network. It is computed as the fraction of closed triads (triangles) over all triads (open and closed). Open triads are 3 nodes in which 2 are connected. Transitivity is defined as:

$\zeta = 3 \times \frac{\text{Number of triangles}}{\text{Number of triads}}$

Assortativity. Assortativity (or degree correlation) is used to quantify the extent of nodes tending to connect to other nodes having similar degree. It can be defined as follows:

$k_{nn}(k) = \frac{\sum_{ij}(a_{ij} - k_i k_j / 2|E|) k_i k_j}{k_i \delta_{ij} - k_i k_j / 2|E|) k_i k_j}$

where δ_{ij} is the Kronecker delta which is 1 if node i connects to node j, 0 otherwise.

Average Distance. Average distance is the average shortest path lengths between all pairs of node in a network. It is defined as follows:

$< d >= \frac{\sum_{i \neq j} d(i,j)}{N(N-1)}$

where $d(i,j)$ is the shortest distance between node i and node j and N is the total number of nodes.

Diameter. The diameter of a network is simply the longest shortest path between all pairs of nodes in a network. It aims to show how far to travel to cover all the network.

Efficiency. Efficiency[11] designates the concept of "small world" property of a network by characterizing how efficiently it exchanges information. The global efficiency is based on averaging the multiplicative inverse of the shortest paths between all nodes.

Degree Distribution. Real-world networks often manifest a scale-free distribution[12]. This characteristic implies the degree distribution tends to follow a power-law. It is well approximated with:

$P(k) = k^{-\gamma}$

where k is the degree of the node, $P(k)$ is the subsequent distribution of the degrees among the nodes, and exponent γ usually falls in the range of [2,3][10].

Mesoscopoic Topological Properties

Mesoscopic characteristics are extracted based on the community structure of a network[13–15]. They can be divided into two types. The first type directly quantifies the overall community structure (such as the mixing parameter and modularity), while the second type is calculated for each community in a network then averaged over all the communities. We denote the second type as $f(c)$. In this paper, nine mesoscopic metrics are used:

Mixing Parameter. The mixing parameter shapes up the fraction of inter-community links. Its value is in the range of [0,1]. The higher the mixing parameter, the more inter-community links, the weaker the community strength is. On the other hand, with a low mixing parameter, networks exhibit a strong community structure due to the existence of only few inter-community links. It is defined as follows:

$\mu = \frac{\sum_{i=1}^{N} k_i^{inter}}{\sum_{i=1}^{N} k_i}$

where k_i^{inter} and $\sum_{i=1}^{N} k_i$ represent the inter-community links of node i and the total degrees in the network, respectively.

Modularity. Modularity measures the quality of community structure. It compares the edge density in each community of a given network with the random version of the same network. Random networks lack a well-defined community structure. If the connections between nodes in a community are higher than random connections, modularity is high. It is defined as follows:

$Q = \sum_{c=1}^{N_c} \left[\frac{m_c}{m} - \left(\frac{k_c}{2m} \right)^2 \right]$

where c is a given community, N_c is the total number of communities, m is the total number of links given by $m = \frac{1}{2} \sum_{i=0}^{N} k_i$, m_c is the total number of links inside community c, k_c is the total number of links inside community c based random connections obtained by the configuration model with the same degree sequence as the original network ($\frac{k_i k_j}{2m}$).

Internal distance. The internal distance is the average distance (or shortest paths) of nodes inside a given community c. It is defined as follows:

$f(c) = \sum_{i,j \in c} \frac{d(i,j)}{n_c(n_c-1)}$

where c is a given community, $d(i,j)$ is the shortest path from node i to node j, n_c is the total number of nodes inside community c.

Internal density. The internal density is the edge density inside a given community c. Averaging the internal density over all communities results in the average internal density of a given network. It is defined as follows:

$f(c) = \frac{2m_c}{n_c(n_c-1)}$

where c is a given community, m_c and n_c are the total number of links and nodes inside community c, respectively.

Maximum-Out Degree Fraction (Max-ODF). Max-ODF is based on the inter-community links of a node that possesses the highest inter-community links in its community c. It is defined as follows:

$f(c) = max_{(i \in c)} \frac{k_i^{inter}}{k_i^{tot}}$

where k_i^{inter} and k_i^{tot} represent the inter-community links and the total degree of node i, respectively.

Average-Out Degree Fraction (Average-ODF). Average-ODF is based on the inter-community links of all the nodes in the community c they belong to. It is defined as follows:

$f(c) = \frac{1}{n_c} \sum_{i \in c} \frac{k_i^{inter}}{k_i^{tot}}$

where n_c is the number of nodes in community c, k_i^{inter} and k_i^{tot} represent the inter-community links and the total degree of node i, respectively.

Flake-Out Degree Fraction (Flake-ODF). Flake-out ODF is based on the percentage of nodes in community c that have more inter-community links than intra-community links. It is defined as follows:

$f(c) = \sum_{i \in c} \frac{|f_i|}{n_c}$

where f_i is the fraction of nodes having $k_i^{inter} \geq k_i^{intra}$ and n_c is the number of nodes in community c.

Embeddedness. Embeddedness quantifies the intra-community links of a node (neighbors in its own community). In fact, it is the opposite of Average-ODF. It reaches a value of 1 if the nodes in a given community c only have intra-community links (all neighbors are in the same community). It is defined as follows:

$f(c) = \frac{1}{n_c} \sum_{i \in c} \frac{k_i^{intra}}{k_i^{tot}}$

where n_c is the number of nodes in community c, k_i^{intra} and k_i^{tot} represent the intra-community links and the total degree of node i, respectively.

Hub Dominance. Hub Dominance is based on the intra-community links of a node that has the highest intra-community links in its community c. It is not the opposite of Max-ODF, as the division here is based on the highest possible degree that can exist in community c, not its total degree (k_i^{tot}). It is defined as follows:

$f(c) = max_{(i \in c)} \frac{k_i^{intra}}{n_c(n_c-1)}$

where k_i^{intra} represent the intra-community links and n_c is the number of nodes in community c.

Supplementary Note 3: Evaluation Measures

Multiple evaluation measures are used in order to uncover the relationship between classical and community-aware centrality measures. They are defined as follows:

Kendall's Tau Correlation

The Kendall's Tau correlation coefficient is usually used to measure the ranking consistency of two sets of nodes. Let's consider two ranked sets $X = (x_1, x_2, .., x_N)$ and $Y = (y_1, y_2, ..., y_N)$ of size N. The pair of ranks (x_i, y_i) and (x_j, y_j) are considered concordant if $x_i > x_j$ and $y_i > y_j$, or if $x_i < x_j$ and $y_i < y_j$. It is said discordant if $x_i > x_j$ and $y_i < y_j$, or if $x_i < x_j$ and $y_i > y_j$. In case of ties (if $x_i = x_j$ or $y_i = y_j$), the pair of ranks is neither concordant nor discordant. The Kendall's Tau correlation τ_b between two ranking sets X and Y of size t is given by:

$$\tau_b(X,Y) = \frac{N_{conc} - N_{disc}}{\sqrt{(N_{conc} + N_{disc} + x)(N_{conc} + N_{disc} + y)}} \quad (1)$$

where N_{conc} and N_{disc} stand for the number of concordant and discordant pairs, respectively, while x and y hold the number of tied pairs in sets X and Y, respectively. The value of τ_b falls in the interval [-1,1]. There is a positive monotonic association between two sets ($\tau_b > 0$), if the values of the two vectors tend to increase or decrease simultaneously. In addition, there is a negative monotonic association between two sets ($\tau_b < 0$), if the values of one vector tend to increase when the values of the other decrease. When τ_b is equal to 0, there is an absence of a monotonic association.

Linear Regression

Linear regression is a statistical approach used to determine the relationship between one dependent variable and one or many independent variables. When only one independent variable is understudy, it is referred to as "simple linear regression". Often, models are fitted using the least squares method to approximate the parameter estimation of each independent variable. In this study, ordinary least squares and weighted least squares methods are used.

Simple Linear Regression. Given a dataset of variables (x_i, y_i) where $i = \{1, 2, ..., M\}$, a simple linear regression model can be defined as:

$$y_i = \phi_0 + \phi_1 x_i + \varepsilon_i$$

where x_i is the independent variable, y_i is the dependent variable, ε_i is the error term, ϕ_0 is the y-intercept, and ϕ_1 is the coefficient of the dependent variable. In our study, x_i refers to the macroscopic and mesoscopic topological properties considered separately for each network, y_i represents the mean of the correlation between classical and local/global community-aware centrality measures considered separately for each network, and M=50 networks.

Ordinary Least Squares. In order to estimate the best coefficients of the dependent variables (ϕ), ordinary least squares (OLS) entails minimizing the sum of squared residuals. Residuals are the differences between the actual value and the fitted value of each observation i. Hence, the aim is to minimize the errors. In OLS, the variance of the residuals is constant across all observations (i.e. $var(\varepsilon_i/x_i) = \sigma^2$). Thus, equal weight is given for each observation in the fitting process. The minimizing criterion can be defined as:

$$\sum_{i=1}^{N} \hat{\varepsilon}_i^{\,2} = \sum_{i=1}^{N} (y_i - \hat{y}_i) = \sum_{i=1}^{N} (y_i - \hat{\phi}_0 - \hat{\phi}_1 x_i)$$

where the y-intercept is obtained by $\hat{\phi}_0 = \bar{y} - \hat{\phi}_1 \bar{x}$, and the slope is obtained by $\hat{\phi}_1 = \frac{\sum_{i=1}^{N}(x_i-\bar{x})(y_i-\bar{y})}{\sum_{i=1}^{N}(x_i-\bar{x})^2}$. Here $\bar{x}$ and $\bar{y}$ are the mean values of variables y and x, respectively. For more details about the derivations of the OLS, one can refer to Wooldridge[16].

Weighted Least Squares. In weighted least squares (WLS), the variance of residuals varies with respect to the observations' variances (i.e. $var(\varepsilon_i/x_i) = \sigma^2 h(\mathbf{x}_i)$). As a result, observations with higher error variance are weighted less than observations with lower error variance in the fitting process. The form of the variance ($h(\mathbf{x}_i)$) of the errors is unknown but can be estimated from the sample set using the method of "feasible generalized least squares"[16] in 5 steps:

1) Run a regression with OLS on the sample set and acquire the estimated residuals $\hat{\varepsilon}_i$
2) Transform the estimated residuals obtained using $\log(\hat{\varepsilon}_i^{\,2})$
3) Run a regression with OLS on the sample set with $\log(\hat{\varepsilon}_i^{\,2})$ as the dependent variable to obtain new estimated coefficients ($\hat{g}_i$)
4) Obtain the weights $\hat{h}_i$ by exponentiating the coefficients ($\hat{h}_i = \exp(\hat{g}_i)$)
5) Divide the original equation of OLS in step 1 by the weights obtained ($\hat{h}_i$) and run a regression with OLS

Infomap and Louvain Community Detection Algorithms

The Infomap algorithm[17] unveils community structure by minimizing the map equation, a partition quality measure based on entropy. It exploits the concept of data compression for a random walker. The random walker first explores the network using its Markov transition matrix. Since most real-world networks exhibit a modular structure, random walkers tend to stay longer inside communities compared to the frequency of moving from one community to another. Codewords can then be used as prefixes for each community. Then, codewords for each node in a given community are assigned (which can be reused across different communities). When the random walker is inside the same community, prefix codes used are minimal. Jumping from one community to another, prefix codes increase. The goal is to build a code that detects communities such that the shortest description for the random walker is acquired. The Louvain algorithm[18] is fundamentally different from Infomap. It is based on optimizing the modularity of a network. It consists of two iterative steps. First, communities are uncovered by maximizing local modularity between the nodes. Second, a new network is built consisting of nodes as the communities found in the previous step, and modularity is maximized on this new network until no further gain can be achieved.

Supplementary Note 4: Real-world Datasets Description

A set of 50 real-world networks is collected from different fields (social, biological, ecological, infrastructure, and collaboration networks)[19–25]. Their size ranges from tens to thousands of nodes and edges. Their macroscopic and mesoscopic characteristics are reported in Table 1 and Table 2, respectively.

Ecological Networks. In the Dolphins network[19], dolphins are connected if there are frequent associations between them. For the Reptiles network (reptilia-tortoise-network)[20], nodes representing tortoises are connected if they use the same refuge hole.

Collaboration Networks. In the NetSci (ca-netscience), GrQc (ca-GrQc), and AstroPh (ca-AstroPh) networks[20], nodes are researchers and edges represent co-authorship of scientific papers based on the researchers specified fields. In the CS Ph.D. network (ca-CSphd)[20], nodes are Ph.D. students and their supervisors specializing in the Computer Science field and edges represent passing scientific knowledge between both. In New Zealand Collaboration (new_zealand_collab)[24], nodes are

institutions (universities, labs, organizations, etc.) and edges represent collaborative publications between the authors in these institutions. In DBLP (dblp_cite)[24], nodes are publications present in the DBLP computer science bibliography and edges represent citations between two given publications.

Human Networks. In the Zachary Karate Club network[25], members are connected if they interact outside the club. In the Jazz network[25], musicians are connected if they have played together in a band. In the Madrid Train Bombings network[25], terrorists are connected if they have contacted each other preparing for the attack on March 11, 2004. For the Adolescent Health network[25], nodes represent students and edges represent both students who chose each other as friends after they have been asked to list 5 of their female and male friends. In Board of Directors (board_directors)[24], nodes represent directors sitting on boards of Norwegian public limited companies and edges represent co-memberships between the directors.

Biological Networks. In the Mouse Visual Cortex network (bn-mouse-visual-cortex-2)[20], nodes are the neurons in the visual cortex of the brain and edges represent the fiber tracts that connect them. In the E. coli Transcription network[22], nodes are Escherichia coli bacteria regulating the conversion of DNA to RNA and edges represent transcriptions between the genes. In Yeast Protein (bio-yeast-protein-inter)[20], Human protein[25], Yeast Collins (collins_yeast)[24], and Interactome Vidal (interactome_vidal)[24], nodes are proteins connected to each other if there's a direct physical interchange. In Budapest Connectome (budapest_connectome)[24], nodes represent brain regions and edges represent the connections between them. In Blumenau Drug (blumenau_drug)[24], nodes represent drugs extracted from electronic health records and edges represent the interactions between them. In Malaria Genes (malaria_genes)[24], nodes are var genes and edges represent a substring shared between two given genes. In Kegg Metabolic (kegg_metabolic)[24], nodes represent substances concerned with enzymatic reactions and edges represent a reaction-production relationship between the given enzymes.

Miscellaneous Networks. In the PolBooks network[25], nodes represent books about U.S. politics and edges represent co-purchases among these books. In the Football network[25], nodes represent American football teams and edges represent games between them during the regular season of Fall 2000. In the Les Misérables network[25], nodes represent actors in Victor Hugo's novel and are connected if they appear in the same chapter of the 'Les Misérables' novel. In the Internet Autonomous Systems (AS-20000102)[22], nodes are autonomous systems (AS) and edges represent connections for exchanging information between two AS. The network is a snapshot of the Internet on January 2, 2000. In Game of Thrones (game_thrones)[24], nodes represent the characters in the Game of Thrones' book "A Storm of Swords" and edges represent their co-appearances if they appeared within 15 words in a given text. In Movie Galaxies (moviegalaxies)[24], nodes are characters in a movie and edges represent co-appearances if actors appeared in the same scene. In Marvel Partnerships (marvel_partnerships)[24], nodes represent either heroes or villains and edges represent partnerships between them. In Internet Topology Cogentco (internet_top_pop)[24], nodes represent countries of the internet graph at the Point of Presence (PoP) level, which is between the IP and AS levels, and edges represent connections between the PoPs. In DNC Emails (dnc)[24], nodes represent members of the Democrat National Committee and edges represent email exchanges between them. In Bible Nouns (bible_nouns)[24], nodes represent noun phrases in the Bible of King James and edges represent co-occurrence if they appear in the same Bible verse. In 911AllWords (911_days_all_words)[21], nodes represent words that appeared in Reuters news for 66 days after the terrorist attacks of September 11 and edges represent the co-appearance of words in the same sentence.

Infrastructure Networks. In the U.S. States network, (contiguous-usa)[20], nodes are the states of America and edges represent border sharing between two given states. In the U.S. Airports network[25], nodes represent airports in America and are connected if there's a direct flight between two given airports. In the U.S. Power Grid network[25], nodes are either a generator, transformer, or substation in the western states of America and edges represent a power supply line. In the EuroRoad network (inf-euroroad)[20], nodes are European cities and edges represent direct transport connection among cities, either within the same country or not. In the EU Airlines network (eu_airlines)[24], nodes are European airports and connections represent airline routes among them. In London Transport (london_transport)[24], nodes represent London train stations either underground, overground, or docklands light railway and edges between them represent a direct transport link.

Online Social Networks. Six different Facebook networks are used. In Facebook Friends (facebook_friends)[24], Ego Facebook (ego-facebook)[20], Caltech (socfb-Caltech36)[20], Princeton (socfb-Princeton12)[20], and Facebook Organizations (facebook_organizations)[24], nodes are Facebook users and edges represent online friendships. In the Facebook Politician Pages network (fb-pages-politician)[20], nodes represent politician pages from different countries and edges convey mutual likes among them. In the DeezerEU[23] network, a platform for music streaming, nodes are users from European countries and edges represent mutual follower relationships. In the PGP network[25], nodes are web of trust users, sharing information under the Pretty Good Privacy algorithm. In the Retweets Copenhagen (rt-twitter-copen)[20], nodes are Twitter users retweeting when the United Nations conference in Copenhagen about climate change took place and edges represent retweet relationships among users. In Hamsterster[25], an online social pet network, nodes represent users in this platform and edges represent friendships among them.

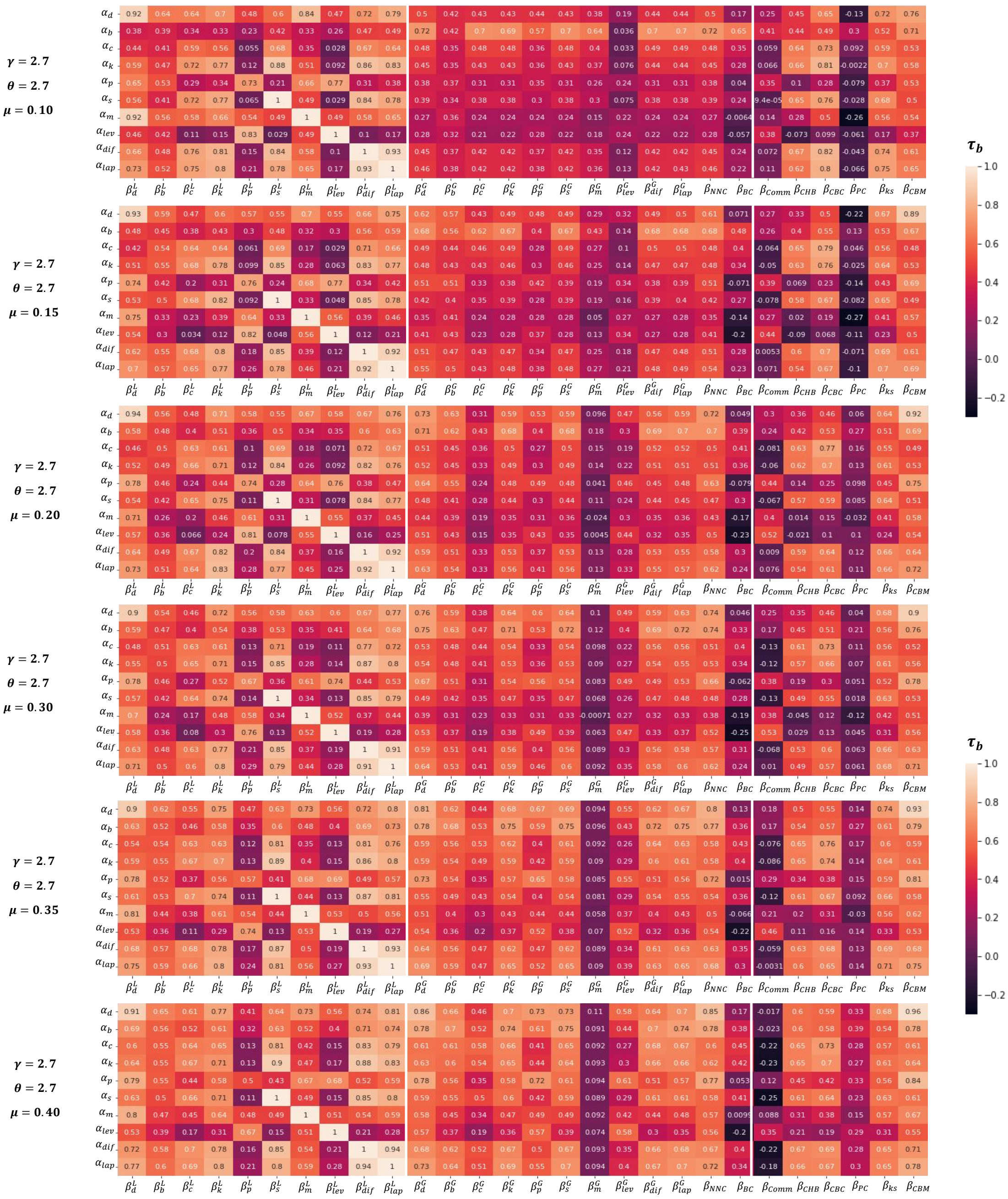

Figure 1. Heatmaps of Kendall's Tau correlation of the various combinations between classical (α_i) and community-aware (β_j) centrality measures in synthetic networks. γ is the exponent of the degree distribution. θ is the exponent of the community size distribution. Networks are sorted in ascending order according to their mixing parameter (μ). The classical centrality measures are: α_d = Degree, α_b = Betweenness, α_c = Closeness, α_k = Katz, α_p = PageRank, α_s = Subgraph, α_m = Maximum Neighborhood Component, α_{lev} = Leverage, α_{dif} = Diffusion, α_{lap} = Laplacian. The local community-aware centrality measures are: (β_d^L, β_b^L, β_c^L, β_k^L ,β_p^L, β_s^L, β_m^L, β_{lev}^L, β_{dif}^L, β_{lap}^L) = the local component of the classical centrality measures based on modular centrality. The global community-aware centrality measures are: (β_d^G, β_b^G, β_c^G, β_k^G ,β_p^G, β_s^G, β_m^G, β_{lev}^G, β_{dif}^G, β_{lap}^G) = the global component of the classical centrality measures based on modular centrality, β_{NNC} = Number of Neighboring Communities centrality, β_{BC} = Bridging centrality. The mixed community-aware centrality measures are: β_{Comm} = Comm centrality, β_{CHB} = Community Hub-Bridge centrality, β_{CBC} = Community-based centrality, β_{PC} = Participation Coefficient, β_{ks} = K-shell with Community centrality, β_{CBM} = Community-based Mediator centrality.

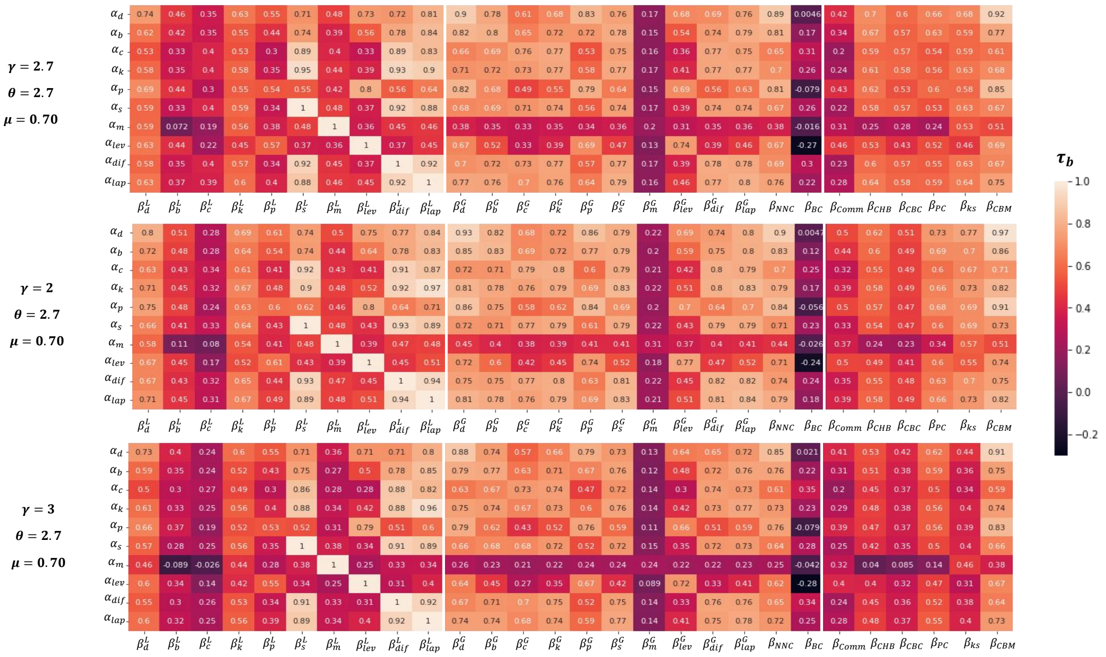

Figure 2. Heatmaps of Kendall's Tau correlation of the various combinations between classical (α_i) and community-aware (β_j) centrality measures in synthetic networks. γ is the exponent of the degree distribution. Three values are used [2, 2.7, 3]. θ is the exponent of the community size distribution, and μ is the mixing parameter. The classical centrality measures are: α_d = Degree, α_b = Betweenness, α_c = Closeness, α_k = Katz, α_p = PageRank, α_s = Subgraph, α_m = Maximum Neighborhood Component, α_{lev} = Leverage, α_{dif} = Diffusion, α_{lap} = Laplacian. The local community-aware centrality measures are: (β_d^L, β_b^L, β_c^L, β_k^L ,β_p^L, β_s^L, β_m^L, β_{lev}^L, β_{dif}^L, β_{lap}^L) = the local component of the classical centrality measures based on modular centrality. The global community-aware centrality measures are: (β_d^G, β_b^G, β_c^G, β_k^G ,β_p^G, β_s^G, β_m^G, β_{lev}^G, β_{dif}^G, β_{lap}^G) = the global component of the classical centrality measures based on modular centrality, β_{NNC} = Number of Neighboring Communities centrality, β_{BC} = Bridging centrality. The mixed community-aware centrality measures are: β_{Comm} = Comm centrality, β_{CHB} = Community Hub-Bridge centrality, β_{CBC} = Community-based centrality, β_{PC} = Participation Coefficient, β_{ks} = K-shell with Community centrality, β_{CBM} = Community-based Mediator centrality.

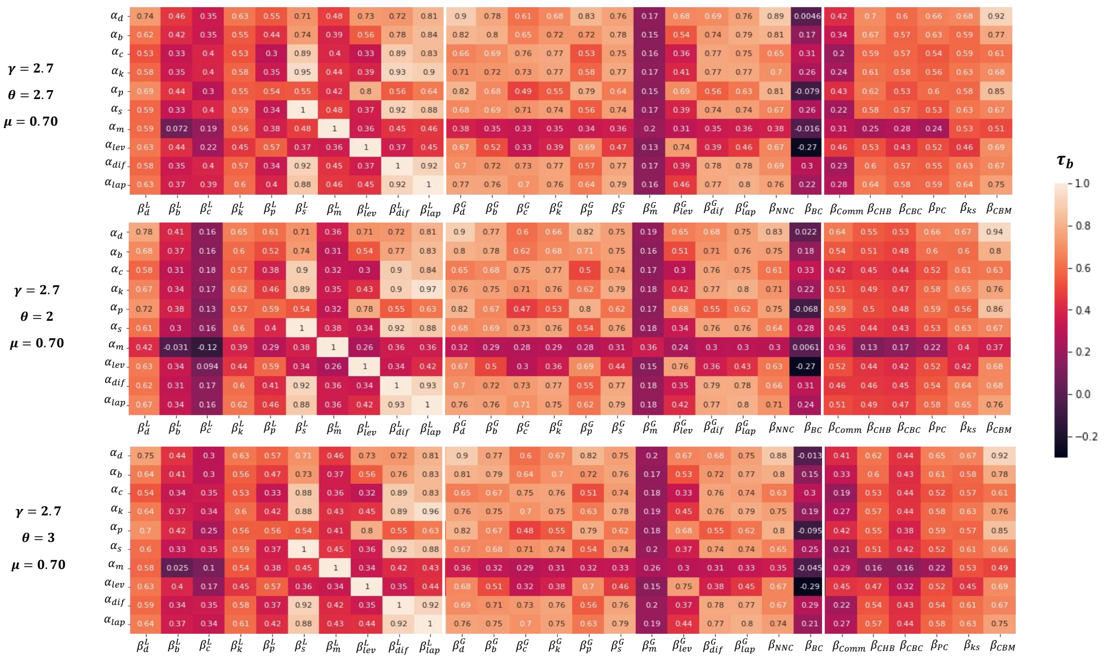

Figure 3. Heatmaps of Kendall's Tau correlation of the various combinations between classical (α_i) and community-aware (β_j) centrality measures in synthetic networks. γ is the exponent of the degree distribution. θ is the exponent of the community size distribution. Three values are used [2, 2.7, 3]. μ is the mixing parameter. The classical centrality measures are: α_d = Degree, α_b = Betweenness, α_c = Closeness, α_k = Katz, α_p = PageRank, α_s = Subgraph, α_m = Maximum Neighborhood Component, α_{lev} = Leverage, α_{dif} = Diffusion, α_{lap} = Laplacian. The local community-aware centrality measures are: (β_d^L, β_b^L, β_c^L, β_k^L ,β_p^L, β_s^L, β_m^L, β_{lev}^L, β_{dif}^L, β_{lap}^L) = the local component of the classical centrality measures based on modular centrality. The global community-aware centrality measures are: (β_d^G, β_b^G, β_c^G, β_k^G ,β_p^G, β_s^G, β_m^G, β_{lev}^G, β_{dif}^G, β_{lap}^G) = the global component of the classical centrality measures based on modular centrality, β_{NNC} = Number of Neighboring Communities centrality, β_{BC} = Bridging centrality. The mixed community-aware centrality measures are: β_{Comm} = Comm centrality, β_{CHB} = Community Hub-Bridge centrality, β_{CBC} = Community-based centrality, β_{PC} = Participation Coefficient, β_{ks} = K-shell with Community centrality, β_{CBM} = Community-based Mediator centrality.

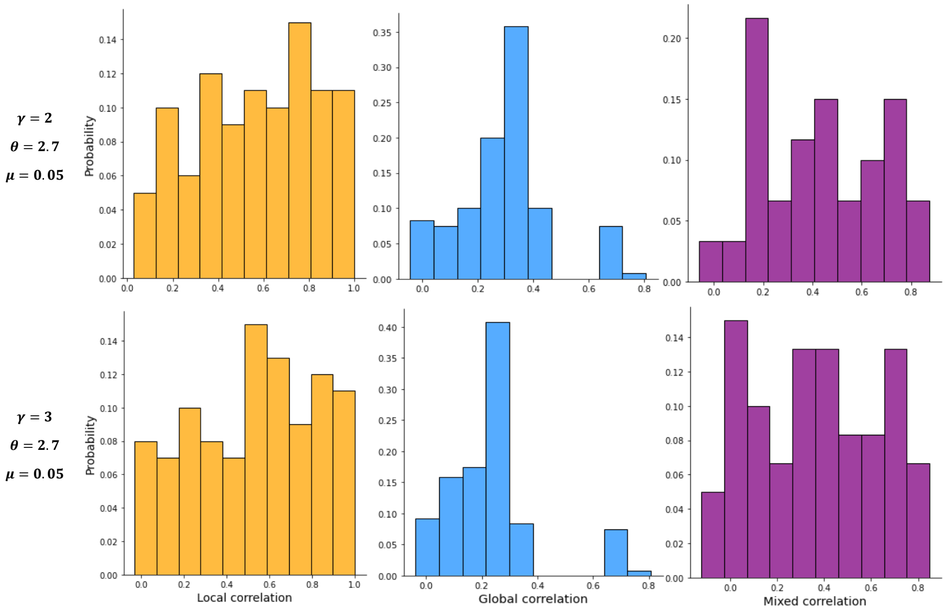

Figure 4. Histograms of the correlation between classical and local, global, and mixed community-aware centrality measures. γ is the exponent of the degree distribution. Two values are used [2, 3]. θ is the exponent of the community size distribution. μ is the mixing parameter.

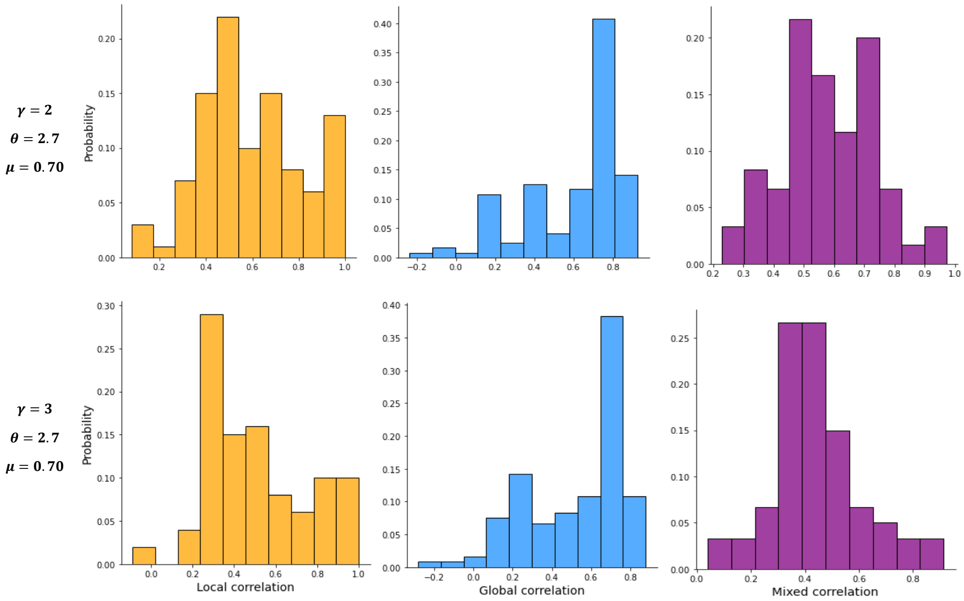

Figure 5. Histograms of the correlation between classical and local, global, and mixed community-aware centrality measures. γ is the exponent of the degree distribution. Two values are used [2, 3]. θ is the exponent of the community size distribution. μ is the mixing parameter.

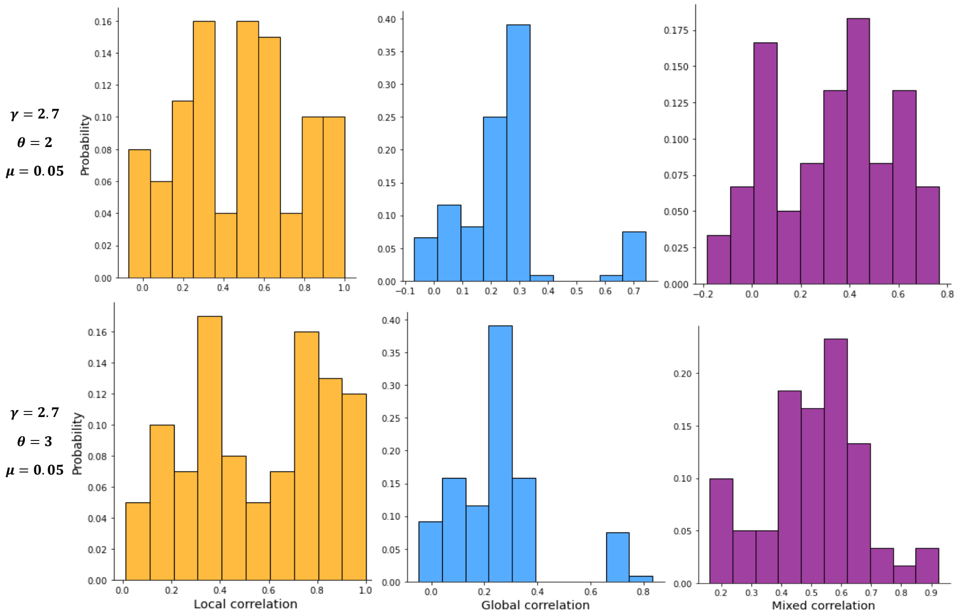

Figure 6. Histograms of the correlation between classical and local, global, and mixed community-aware centrality measures. γ is the exponent of the degree distribution. θ is the exponent of the community size distribution. Two values are used [2, 3]. μ is the mixing parameter.

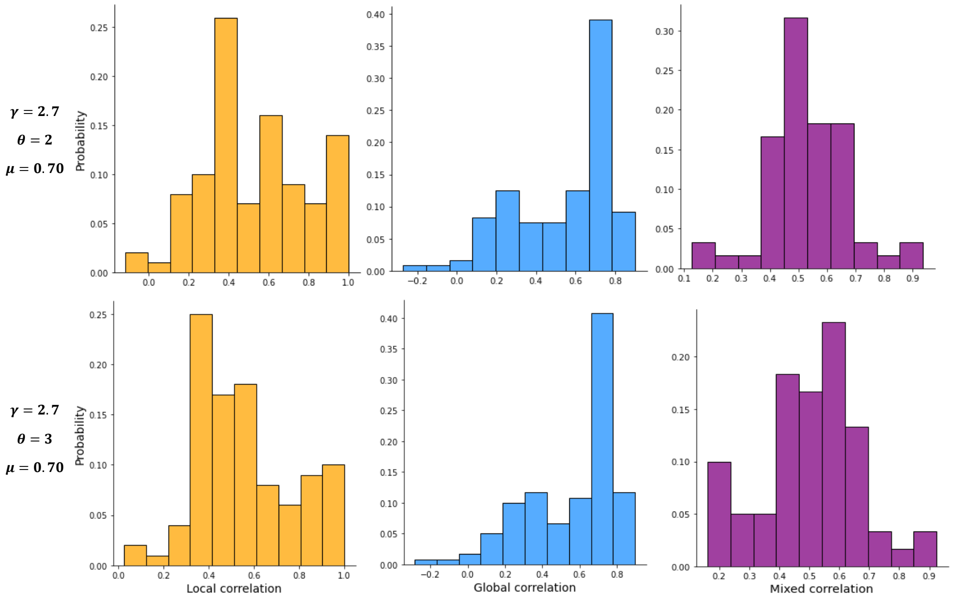

Figure 7. Histograms of the correlation between classical and local, global, and mixed community-aware centrality measures. γ is the exponent of the degree distribution. θ is the exponent of the community size distribution. Two values are used [2, 3]. μ is the mixing parameter.

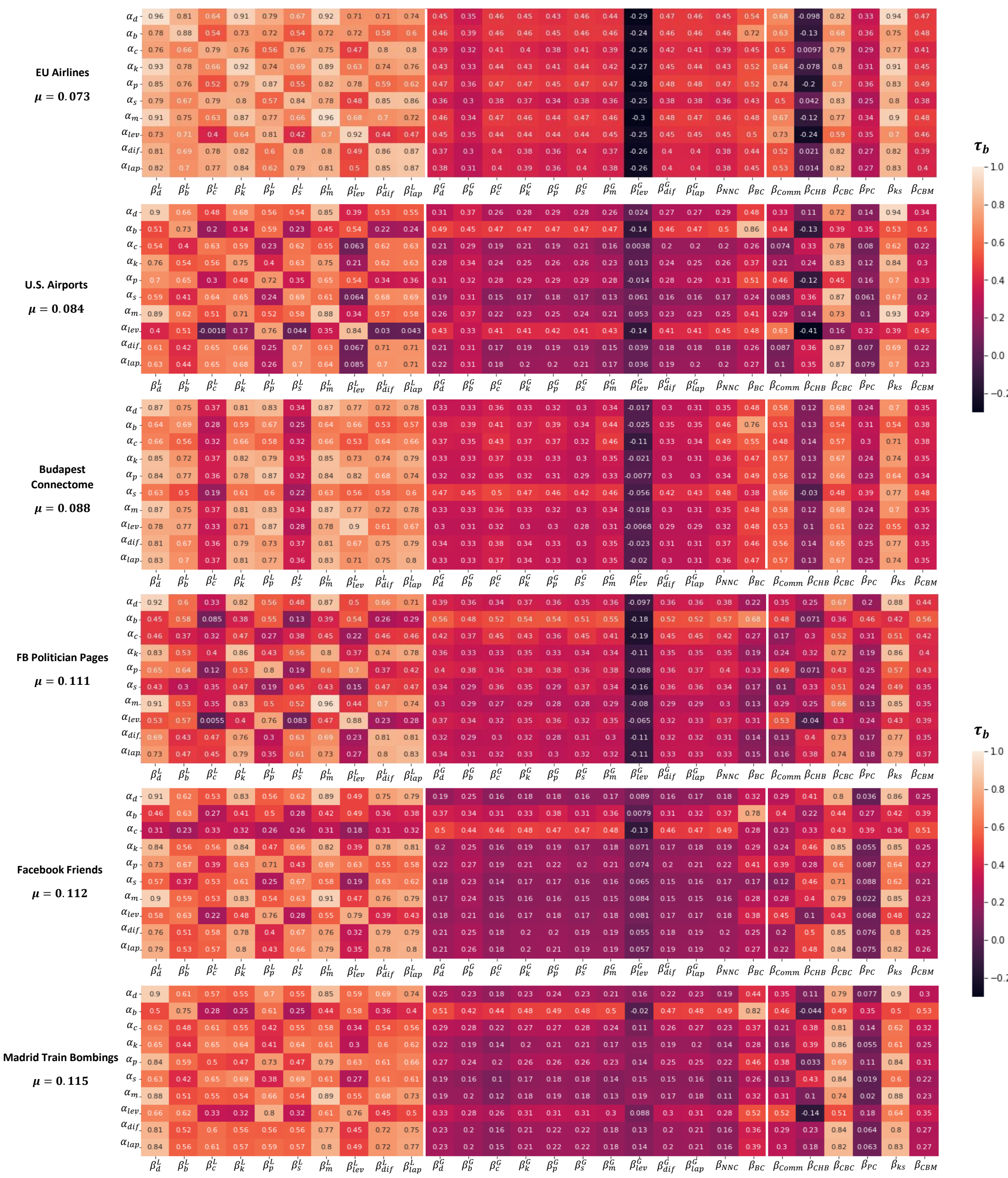

Figure 8. Heatmaps of Kendall's Tau correlation of the various combinations between classical (α_i) and community-aware (β_j) centrality measures in 6 real-world networks. Networks are sorted in ascending order according to their mixing parameter (μ). The mixing parameter is deduced after the community structure is uncovered using Infomap community detection algorithm. The classical centrality measures are: α_d = Degree, α_b = Betweenness, α_c = Closeness, α_k = Katz, α_p = PageRank, α_s = Subgraph, α_m = Maximum Neighborhood Component, α_{lev} = Leverage, α_{dif} = Diffusion, α_{lap} = Laplacian. The local community-aware centrality measures are: (β_d^L, β_b^L, β_c^L, β_k^L ,β_p^L, β_s^L, β_m^L, β_{lev}^L, β_{dif}^L, β_{lap}^L) = the local component of the classical centrality measures based on modular centrality. The global community-aware centrality measures are: (β_d^G, β_b^G, β_c^G, β_k^G ,β_p^G, β_s^G, β_m^G, β_{lev}^G, β_{dif}^G, β_{lap}^G) = the global component of the classical centrality measures based on modular centrality, β_{NNC} = Number of Neighboring Communities centrality, β_{BC} = Bridging centrality. The mixed community-aware centrality measures are: β_{Comm} = Comm centrality, β_{CHB} = Community Hub-Bridge centrality, β_{CBC} = Community-based centrality, β_{PC} = Participation Coefficient, β_{ks} = K-shell with Community centrality, β_{CBM} = Community-based Mediator centrality.

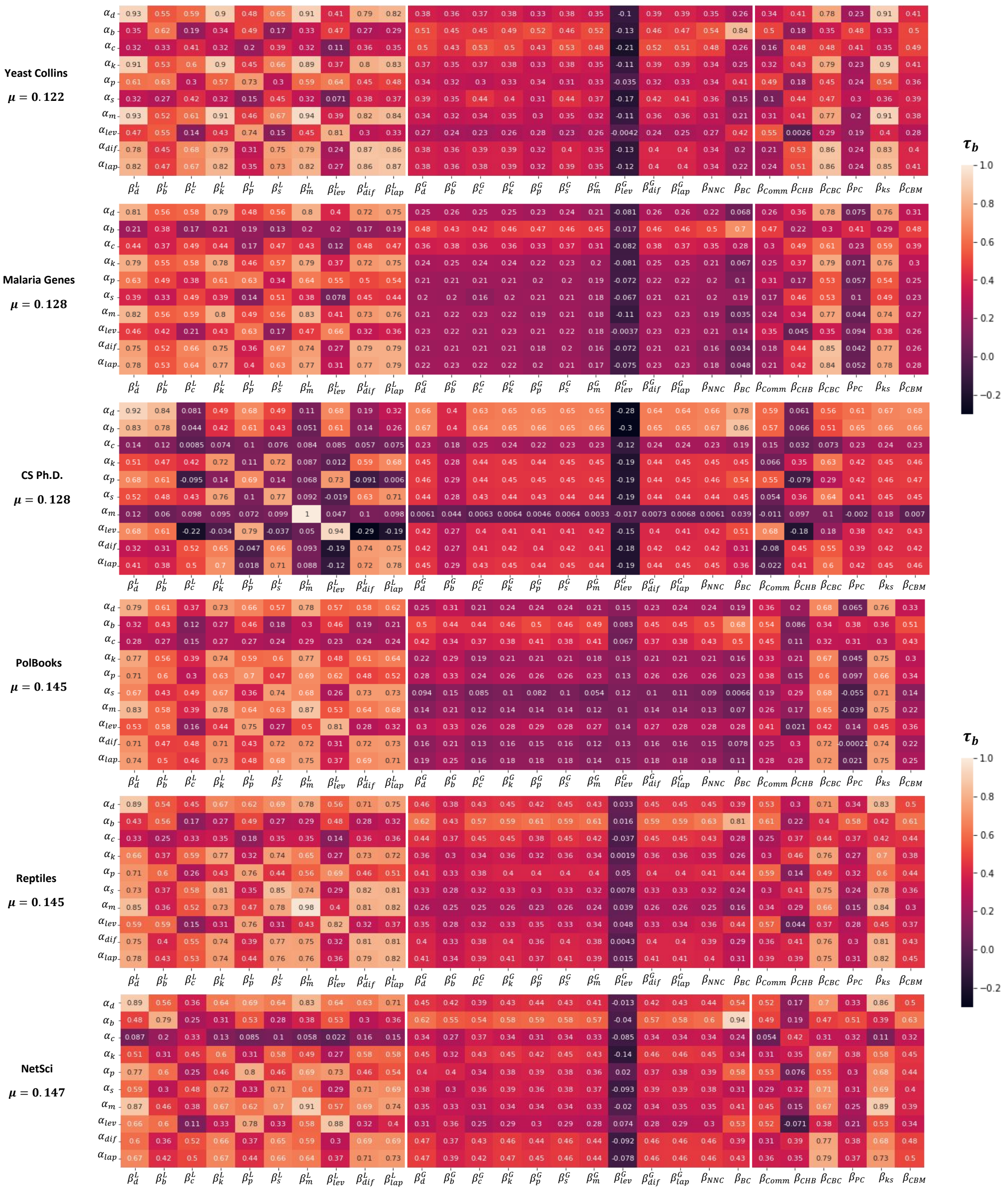

Figure 9. Heatmaps of Kendall's Tau correlation of the various combinations between classical (α_i) and community-aware (β_j) centrality measures in 6 real-world networks. Networks are sorted in ascending order according to their mixing parameter (μ). The mixing parameter is deduced after the community structure is uncovered using Infomap community detection algorithm. The classical centrality measures are: α_d = Degree, α_b = Betweenness, α_c = Closeness, α_k = Katz, α_p = PageRank, α_s = Subgraph, α_m = Maximum Neighborhood Component, α_{lev} = Leverage, α_{dif} = Diffusion, α_{lap} = Laplacian. The local community-aware centrality measures are: (β_d^L, β_b^L, β_c^L, β_k^L ,β_p^L, β_s^L, β_m^L, β_{lev}^L, β_{dif}^L, β_{lap}^L) = the local component of the classical centrality measures based on modular centrality. The global community-aware centrality measures are: (β_d^G, β_b^G, β_c^G, β_k^G ,β_p^G, β_s^G, β_m^G, β_{lev}^G, β_{dif}^G, β_{lap}^G) = the global component of the classical centrality measures based on modular centrality, β_{NNC} = Number of Neighboring Communities centrality, β_{BC} = Bridging centrality. The mixed community-aware centrality measures are: β_{Comm} = Comm centrality, β_{CHB} = Community Hub-Bridge centrality, β_{CBC} = Community-based centrality, β_{PC} = Participation Coefficient, β_{ks} = K-shell with Community centrality, β_{CBM} = Community-based Mediator centrality.

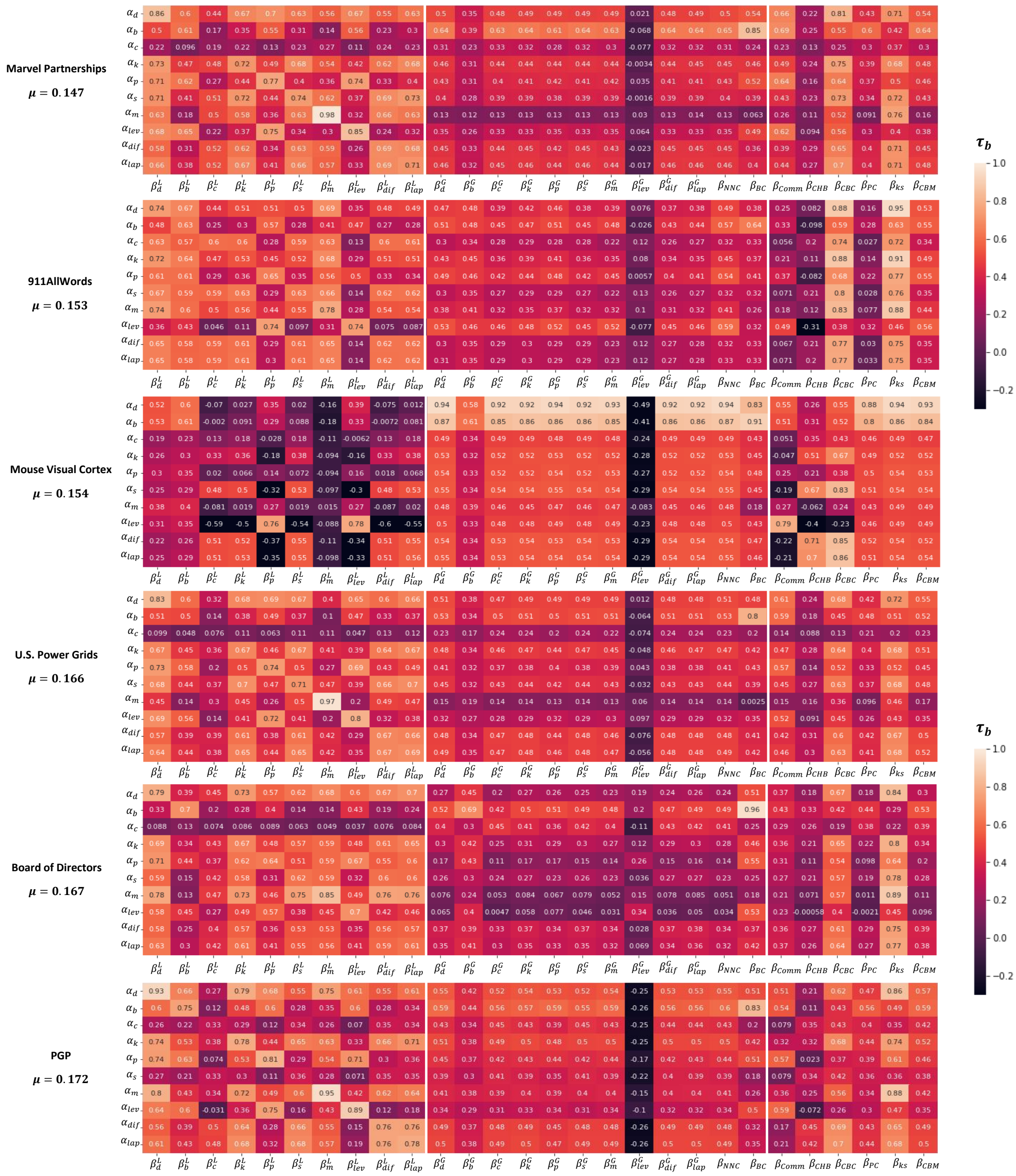

Figure 10. Heatmaps of Kendall's Tau correlation of the various combinations between classical (α_i) and community-aware (β_j) centrality measures in 6 real-world networks. Networks are sorted in ascending order according to their mixing parameter (μ). The mixing parameter is deduced after the community structure is uncovered using Infomap community detection algorithm. The classical centrality measures are: α_d = Degree, α_b = Betweenness, α_c = Closeness, α_k = Katz, α_p = PageRank, α_s = Subgraph, α_m = Maximum Neighborhood Component, α_{lev} = Leverage, α_{dif} = Diffusion, α_{lap} = Laplacian. The local community-aware centrality measures are: (β_d^L, β_b^L, β_c^L, β_k^L ,β_p^L, β_s^L, β_m^L, β_{lev}^L, β_{dif}^L, β_{lap}^L) = the local component of the classical centrality measures based on modular centrality. The global community-aware centrality measures are: (β_d^G, β_b^G, β_c^G, β_k^G ,β_p^G, β_s^G, β_m^G, β_{lev}^G, β_{dif}^G, β_{lap}^G) = the global component of the classical centrality measures based on modular centrality, β_{NNC} = Number of Neighboring Communities centrality, β_{BC} = Bridging centrality. The mixed community-aware centrality measures are: β_{Comm} = Comm centrality, β_{CHB} = Community Hub-Bridge centrality, β_{CBC} = Community-based centrality, β_{PC} = Participation Coefficient, β_{ks} = K-shell with Community centrality, β_{CBM} = Community-based Mediator centrality.

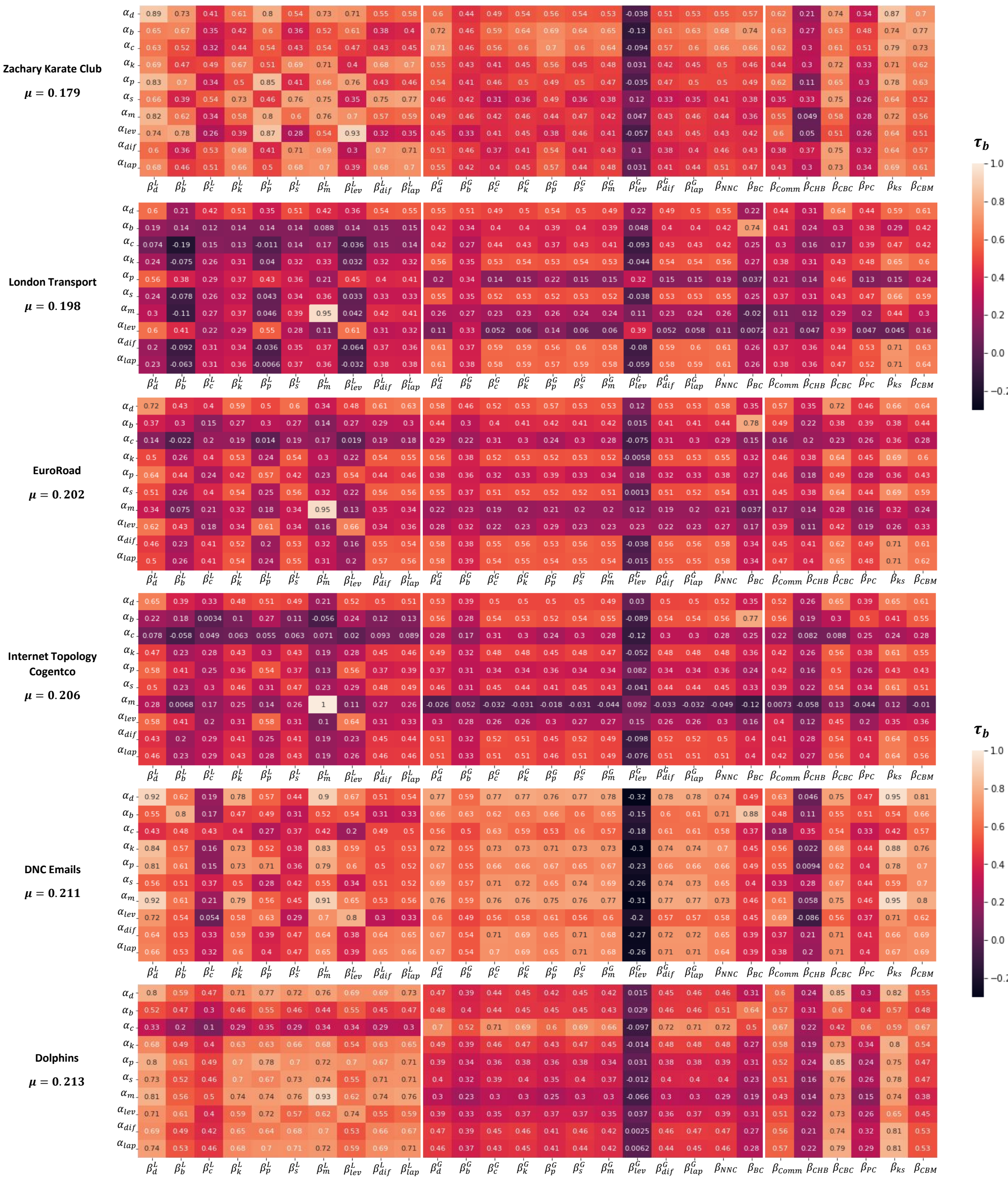

Figure 11. Heatmaps of Kendall's Tau correlation of the various combinations between classical (α_i) and community-aware (β_j) centrality measures in 6 real-world networks. Networks are sorted in ascending order according to their mixing parameter (μ). The mixing parameter is deduced after the community structure is uncovered using Infomap community detection algorithm. The classical centrality measures are: α_d = Degree, α_b = Betweenness, α_c = Closeness, α_k = Katz, α_p = PageRank, α_s = Subgraph, α_m = Maximum Neighborhood Component, α_{lev} = Leverage, α_{dif} = Diffusion, α_{lap} = Laplacian. The local community-aware centrality measures are: (β_d^L, β_b^L, β_c^L, β_k^L ,β_p^L, β_s^L, β_m^L, β_{lev}^L, β_{dif}^L, β_{lap}^L) = the local component of the classical centrality measures based on modular centrality. The global community-aware centrality measures are: (β_d^G, β_b^G, β_c^G, β_k^G ,β_p^G, β_s^G, β_m^G, β_{lev}^G, β_{dif}^G, β_{lap}^G) = the global component of the classical centrality measures based on modular centrality, β_{NNC} = Number of Neighboring Communities centrality, β_{BC} = Bridging centrality. The mixed community-aware centrality measures are: β_{Comm} = Comm centrality, β_{CHB} = Community Hub-Bridge centrality, β_{CBC} = Community-based centrality, β_{PC} = Participation Coefficient, β_{ks} = K-shell with Community centrality, β_{CBM} = Community-based Mediator centrality.

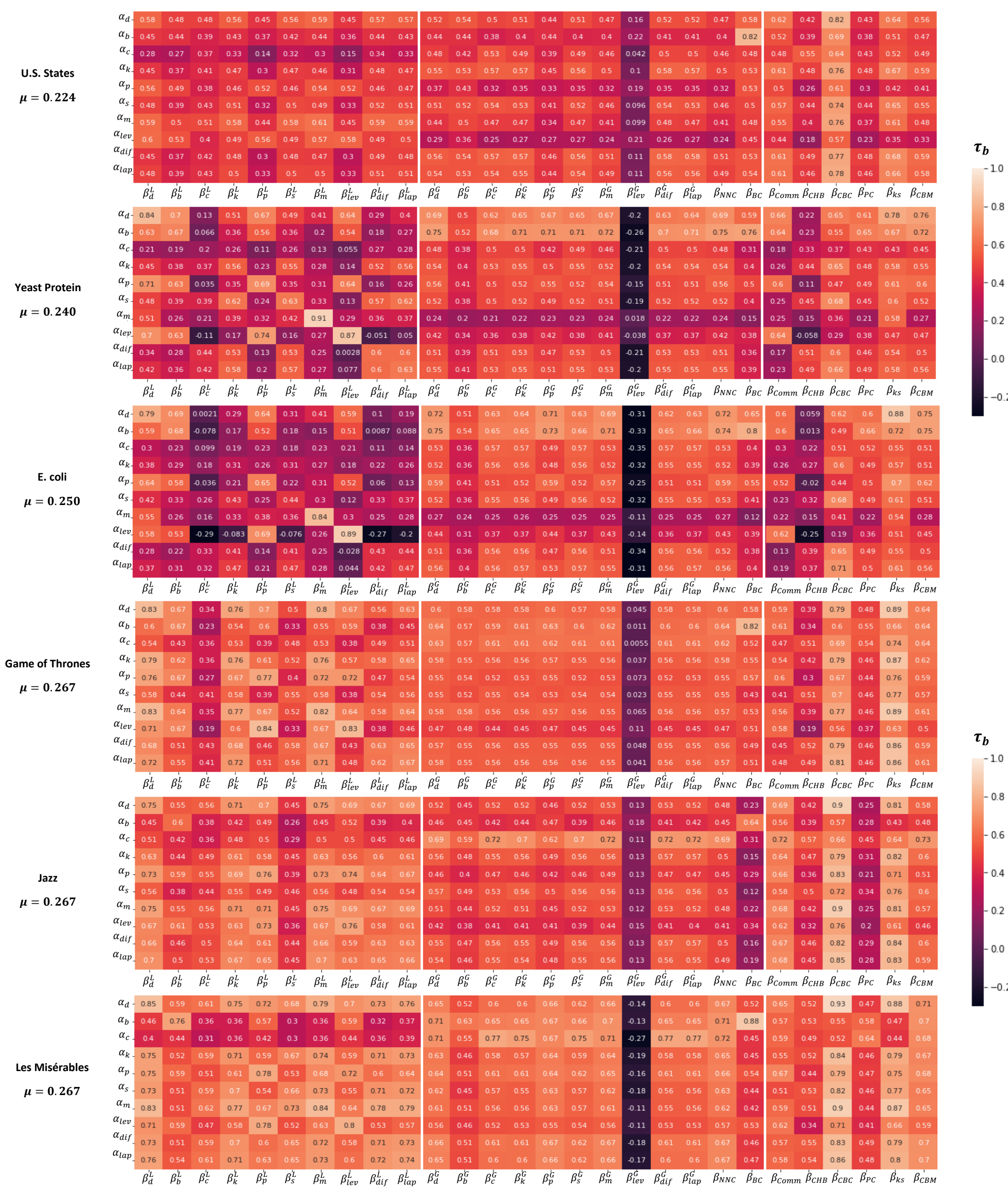

Figure 12. Heatmaps of Kendall's Tau correlation of the various combinations between classical (α_i) and community-aware (β_j) centrality measures in 6 real-world networks. Networks are sorted in ascending order according to their mixing parameter (μ). The mixing parameter is deduced after the community structure is uncovered using Infomap community detection algorithm. The classical centrality measures are: α_d = Degree, α_b = Betweenness, α_c = Closeness, α_k = Katz, α_p = PageRank, α_s = Subgraph, α_m = Maximum Neighborhood Component, α_{lev} = Leverage, α_{dif} = Diffusion, α_{lap} = Laplacian. The local community-aware centrality measures are: (β_d^L, β_b^L, β_c^L, β_k^L ,β_p^L, β_s^L, β_m^L, β_{lev}^L, β_{dif}^L, β_{lap}^L) = the local component of the classical centrality measures based on modular centrality. The global community-aware centrality measures are: (β_d^G, β_b^G, β_c^G, β_k^G ,β_p^G, β_s^G, β_m^G, β_{lev}^G, β_{dif}^G, β_{lap}^G) = the global component of the classical centrality measures based on modular centrality, β_{NNC} = Number of Neighboring Communities centrality, β_{BC} = Bridging centrality. The mixed community-aware centrality measures are: β_{Comm} = Comm centrality, β_{CHB} = Community Hub-Bridge centrality, β_{CBC} = Community-based centrality, β_{PC} = Participation Coefficient, β_{ks} = K-shell with Community centrality, β_{CBM} = Community-based Mediator centrality.

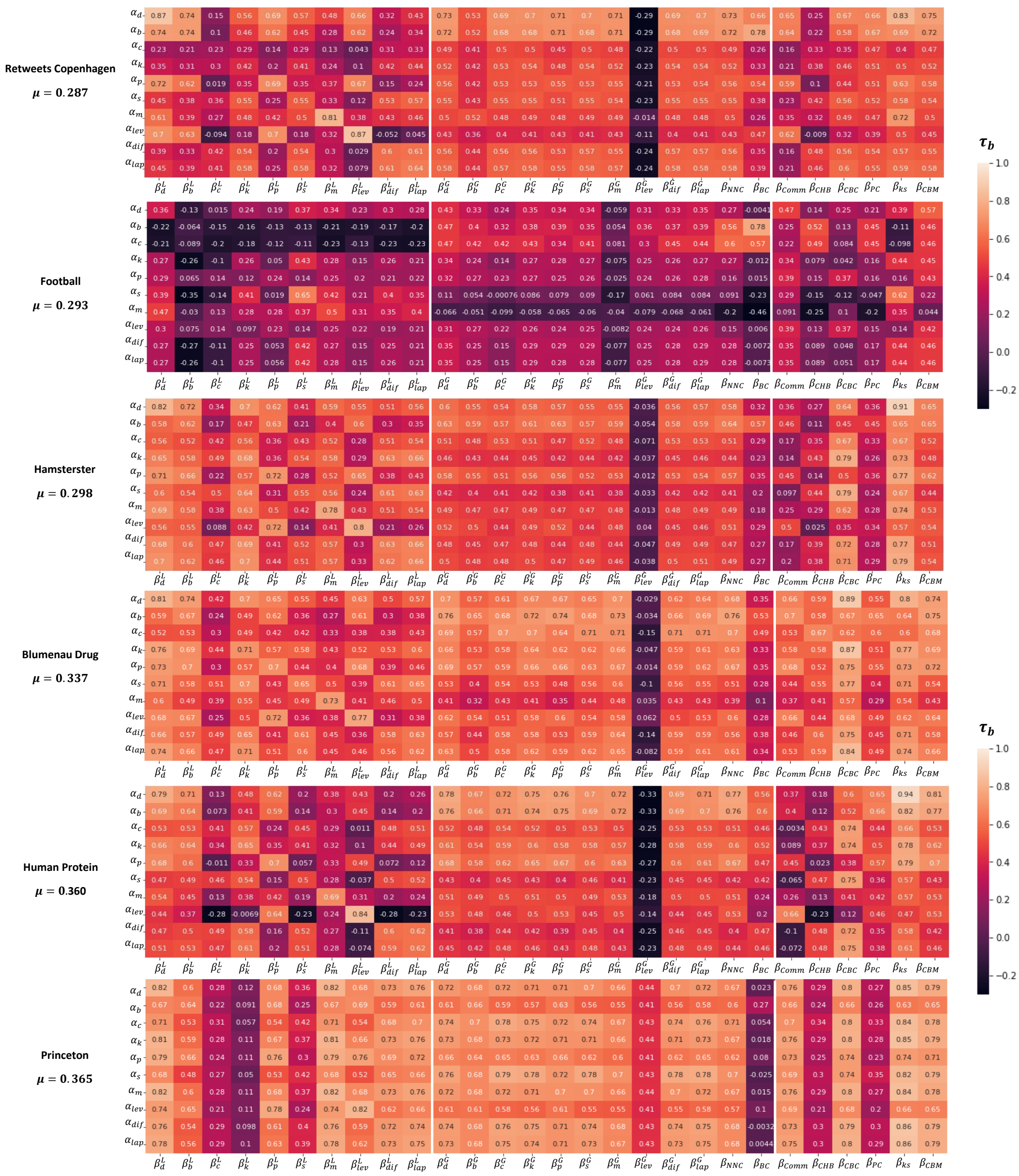

Figure 13. Heatmaps of Kendall's Tau correlation of the various combinations between classical (α_i) and community-aware (β_j) centrality measures in 6 real-world networks. Networks are sorted in ascending order according to their mixing parameter (μ). The mixing parameter is deduced after the community structure is uncovered using Infomap community detection algorithm. The classical centrality measures are: α_d = Degree, α_b = Betweenness, α_c = Closeness, α_k = Katz, α_p = PageRank, α_s = Subgraph, α_m = Maximum Neighborhood Component, α_{lev} = Leverage, α_{dif} = Diffusion, α_{lap} = Laplacian. The local community-aware centrality measures are: (β_d^L, β_b^L, β_c^L, β_k^L ,β_p^L, β_s^L, β_m^L, β_{lev}^L, β_{dif}^L, β_{lap}^L) = the local component of the classical centrality measures based on modular centrality. The global community-aware centrality measures are: (β_d^G, β_b^G, β_c^G, β_k^G ,β_p^G, β_s^G, β_m^G, β_{lev}^G, β_{dif}^G, β_{lap}^G) = the global component of the classical centrality measures based on modular centrality, β_{NNC} = Number of Neighboring Communities centrality, β_{BC} = Bridging centrality. The mixed community-aware centrality measures are: β_{Comm} = Comm centrality, β_{CHB} = Community Hub-Bridge centrality, β_{CBC} = Community-based centrality, β_{PC} = Participation Coefficient, β_{ks} = K-shell with Community centrality, β_{CBM} = Community-based Mediator centrality.

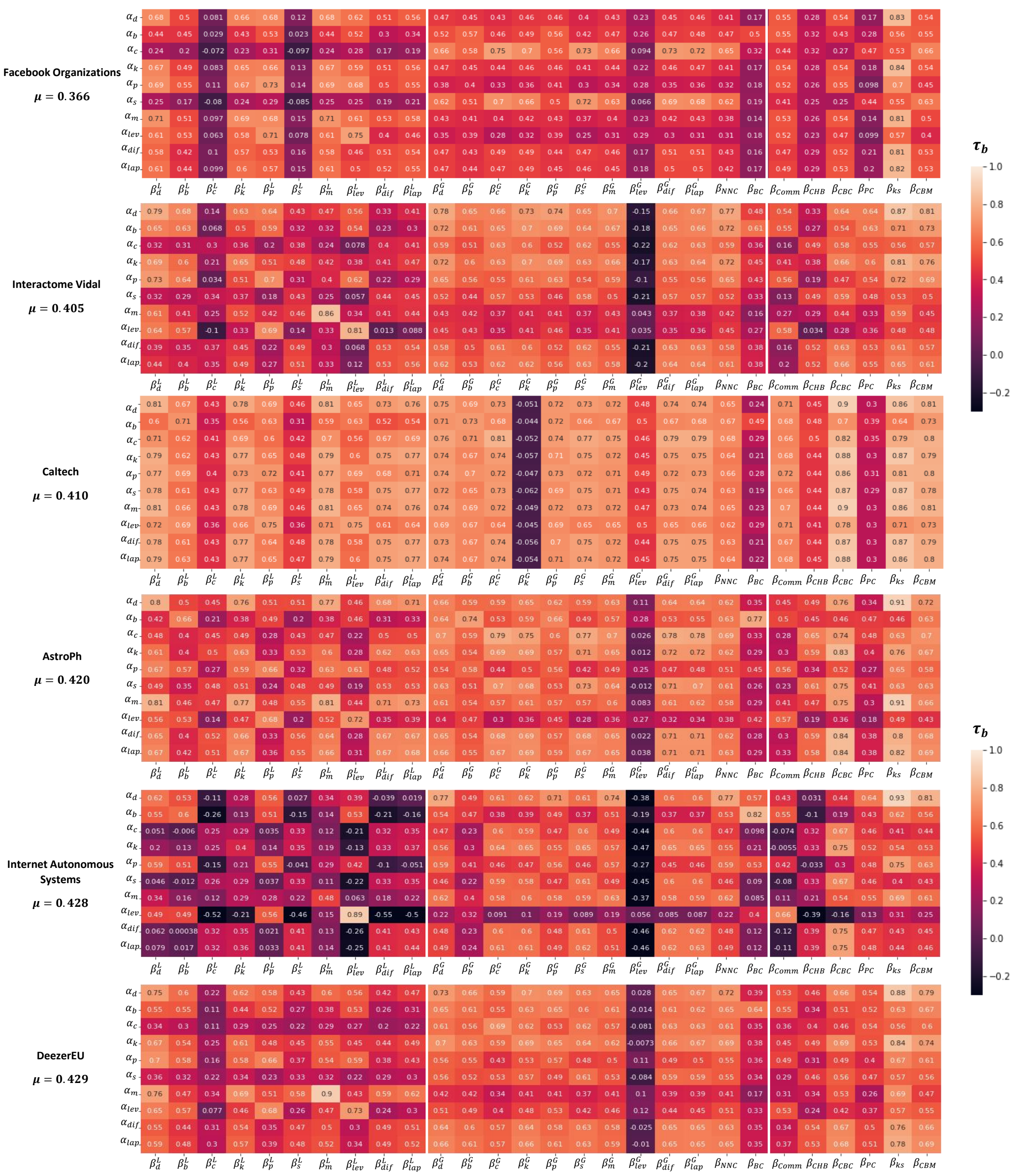

Figure 14. Heatmaps of Kendall's Tau correlation of the various combinations between classical (α_i) and community-aware (β_j) centrality measures in 6 real-world networks. Networks are sorted in ascending order according to their mixing parameter (μ). The mixing parameter is deduced after the community structure is uncovered using Infomap community detection algorithm. The classical centrality measures are: α_d = Degree, α_b = Betweenness, α_c = Closeness, α_k = Katz, α_p = PageRank, α_s = Subgraph, α_m = Maximum Neighborhood Component, α_{lev} = Leverage, α_{dif} = Diffusion, α_{lap} = Laplacian. The local community-aware centrality measures are: (β_d^L, β_b^L, β_c^L, β_k^L ,β_p^L, β_s^L, β_m^L, β_{lev}^L, β_{dif}^L, β_{lap}^L) = the local component of the classical centrality measures based on modular centrality. The global community-aware centrality measures are: (β_d^G, β_b^G, β_c^G, β_k^G ,β_p^G, β_s^G, β_m^G, β_{lev}^G, β_{dif}^G, β_{lap}^G) = the global component of the classical centrality measures based on modular centrality, β_{NNC} = Number of Neighboring Communities centrality, β_{BC} = Bridging centrality. The mixed community-aware centrality measures are: β_{Comm} = Comm centrality, β_{CHB} = Community Hub-Bridge centrality, β_{CBC} = Community-based centrality, β_{PC} = Participation Coefficient, β_{ks} = K-shell with Community centrality, β_{CBM} = Community-based Mediator centrality.

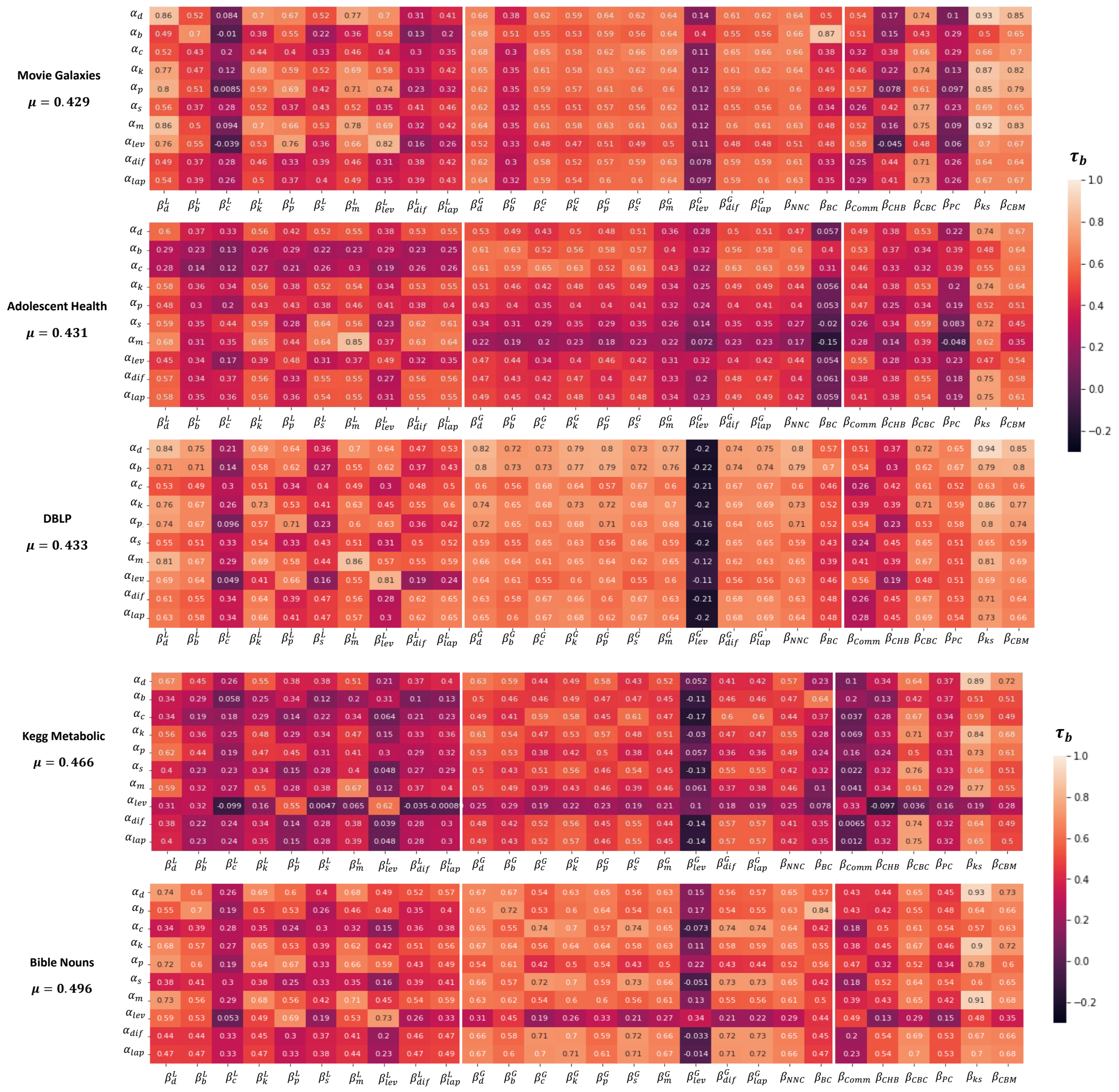

Figure 15. Heatmaps of Kendall's Tau correlation of the various combinations between classical (α_i) and community-aware (β_j) centrality measures in 6 real-world networks. Networks are sorted in ascending order according to their mixing parameter (μ). The mixing parameter is deduced after the community structure is uncovered using Infomap community detection algorithm. The classical centrality measures are: α_d = Degree, α_b = Betweenness, α_c = Closeness, α_k = Katz, α_p = PageRank, α_s = Subgraph, α_m = Maximum Neighborhood Component, α_{lev} = Leverage, α_{dif} = Diffusion, α_{lap} = Laplacian. The local community-aware centrality measures are: (β_d^L, β_b^L, β_c^L, β_k^L ,β_p^L, β_s^L, β_m^L, β_{lev}^L, β_{dif}^L, β_{lap}^L) = the local component of the classical centrality measures based on modular centrality. The global community-aware centrality measures are: (β_d^G, β_b^G, β_c^G, β_k^G ,β_p^G, β_s^G, β_m^G, β_{lev}^G, β_{dif}^G, β_{lap}^G) = the global component of the classical centrality measures based on modular centrality, β_{NNC} = Number of Neighboring Communities centrality, β_{BC} = Bridging centrality. The mixed community-aware centrality measures are: β_{Comm} = Comm centrality, β_{CHB} = Community Hub-Bridge centrality, β_{CBC} = Community-based centrality, β_{PC} = Participation Coefficient, β_{ks} = K-shell with Community centrality, β_{CBM} = Community-based Mediator centrality.

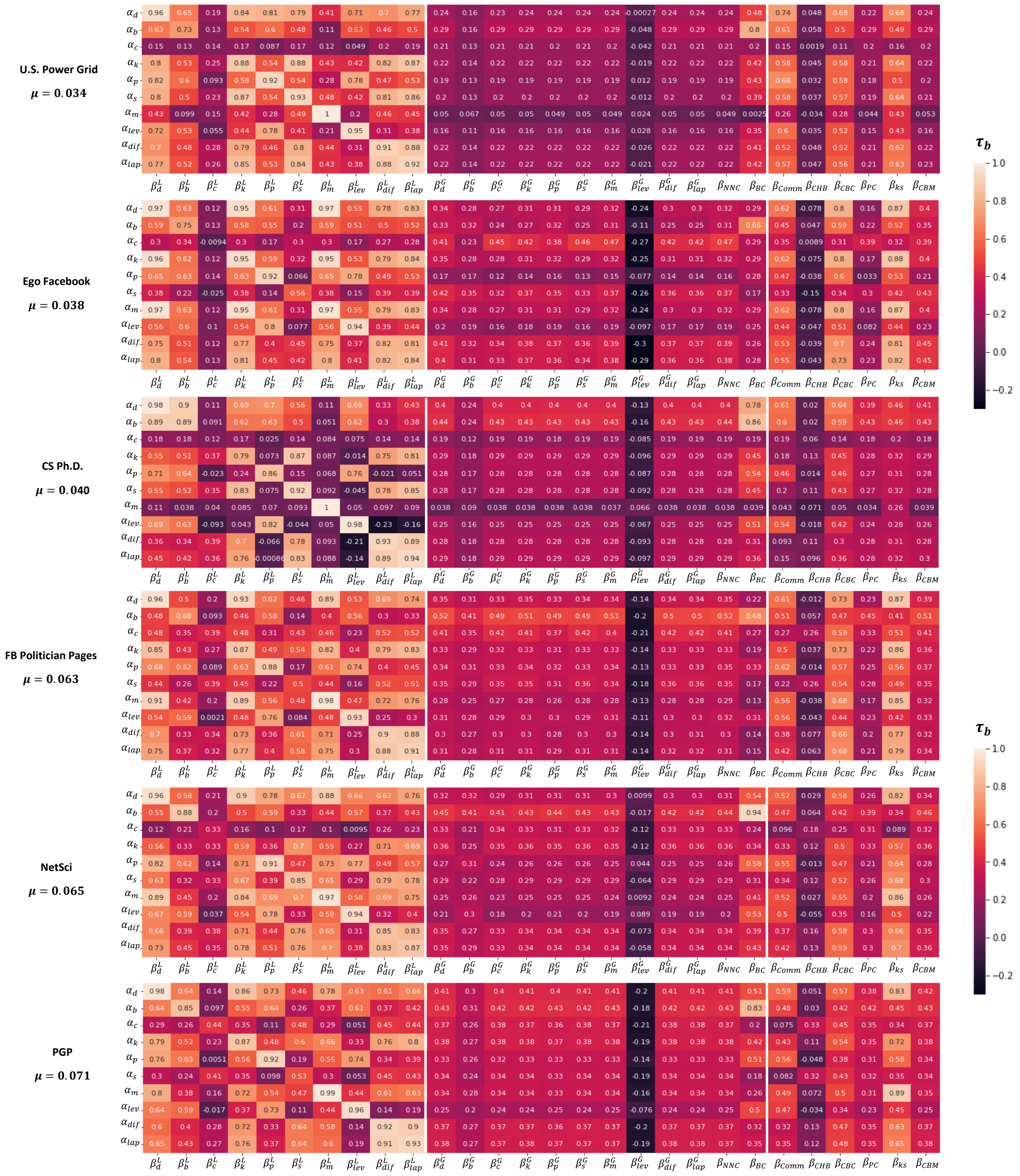

Figure 16. Heatmaps of Kendall's Tau correlation of the various combinations between classical (α_i) and community-aware (β_j) centrality measures in 6 real-world networks. Networks are sorted in ascending order according to their mixing parameter (μ). The mixing parameter is deduced after the community structure is uncovered using Louvain community detection algorithm. The classical centrality measures are: α_d = Degree, α_b = Betweenness, α_c = Closeness, α_k = Katz, α_p = PageRank, α_s = Subgraph, α_m = Maximum Neighborhood Component, α_{lev} = Leverage, α_{dif} = Diffusion, α_{lap} = Laplacian. The local community-aware centrality measures are: (β_d^L, β_b^L, β_c^L, β_k^L ,β_p^L, β_s^L, β_m^L, β_{lev}^L, β_{dif}^L, β_{lap}^L) = the local component of the classical centrality measures based on modular centrality. The global community-aware centrality measures are: (β_d^G, β_b^G, β_c^G, β_k^G ,β_p^G, β_s^G, β_m^G, β_{lev}^G, β_{dif}^G, β_{lap}^G) = the global component of the classical centrality measures based on modular centrality, β_{NNC} = Number of Neighboring Communities centrality, β_{BC} = Bridging centrality. The mixed community-aware centrality measures are: β_{Comm} = Comm centrality, β_{CHB} = Community Hub-Bridge centrality, β_{CBC} = Community-based centrality, β_{PC} = Participation Coefficient, β_{ks} = K-shell with Community centrality, β_{CBM} = Community-based Mediator centrality.

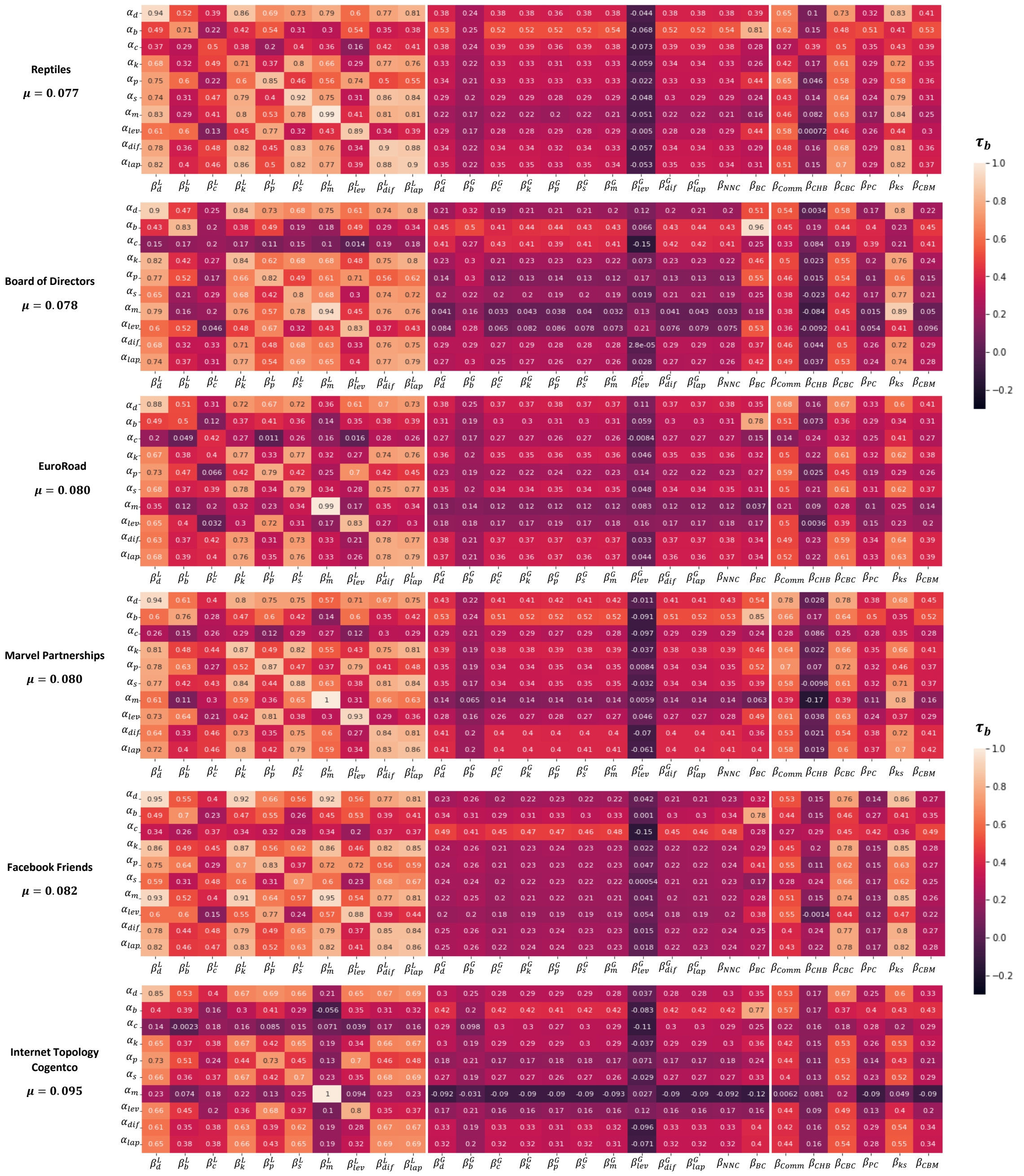

Figure 17. Heatmaps of Kendall's Tau correlation of the various combinations between classical (α_i) and community-aware (β_j) centrality measures in 6 real-world networks. Networks are sorted in ascending order according to their mixing parameter (μ). The mixing parameter is deduced after the community structure is uncovered using Louvain community detection algorithm. The classical centrality measures are: α_d = Degree, α_b = Betweenness, α_c = Closeness, α_k = Katz, α_p = PageRank, α_s = Subgraph, α_m = Maximum Neighborhood Component, α_{lev} = Leverage, α_{dif} = Diffusion, α_{lap} = Laplacian. The local community-aware centrality measures are: (β_d^L, β_b^L, β_c^L, β_k^L ,β_p^L, β_s^L, β_m^L, β_{lev}^L, β_{dif}^L, β_{lap}^L) = the local component of the classical centrality measures based on modular centrality. The global community-aware centrality measures are: (β_d^G, β_b^G, β_c^G, β_k^G ,β_p^G, β_s^G, β_m^G, β_{lev}^G, β_{dif}^G, β_{lap}^G) = the global component of the classical centrality measures based on modular centrality, β_{NNC} = Number of Neighboring Communities centrality, β_{BC} = Bridging centrality. The mixed community-aware centrality measures are: β_{Comm} = Comm centrality, β_{CHB} = Community Hub-Bridge centrality, β_{CBC} = Community-based centrality, β_{PC} = Participation Coefficient, β_{ks} = K-shell with Community centrality, β_{CBM} = Community-based Mediator centrality.

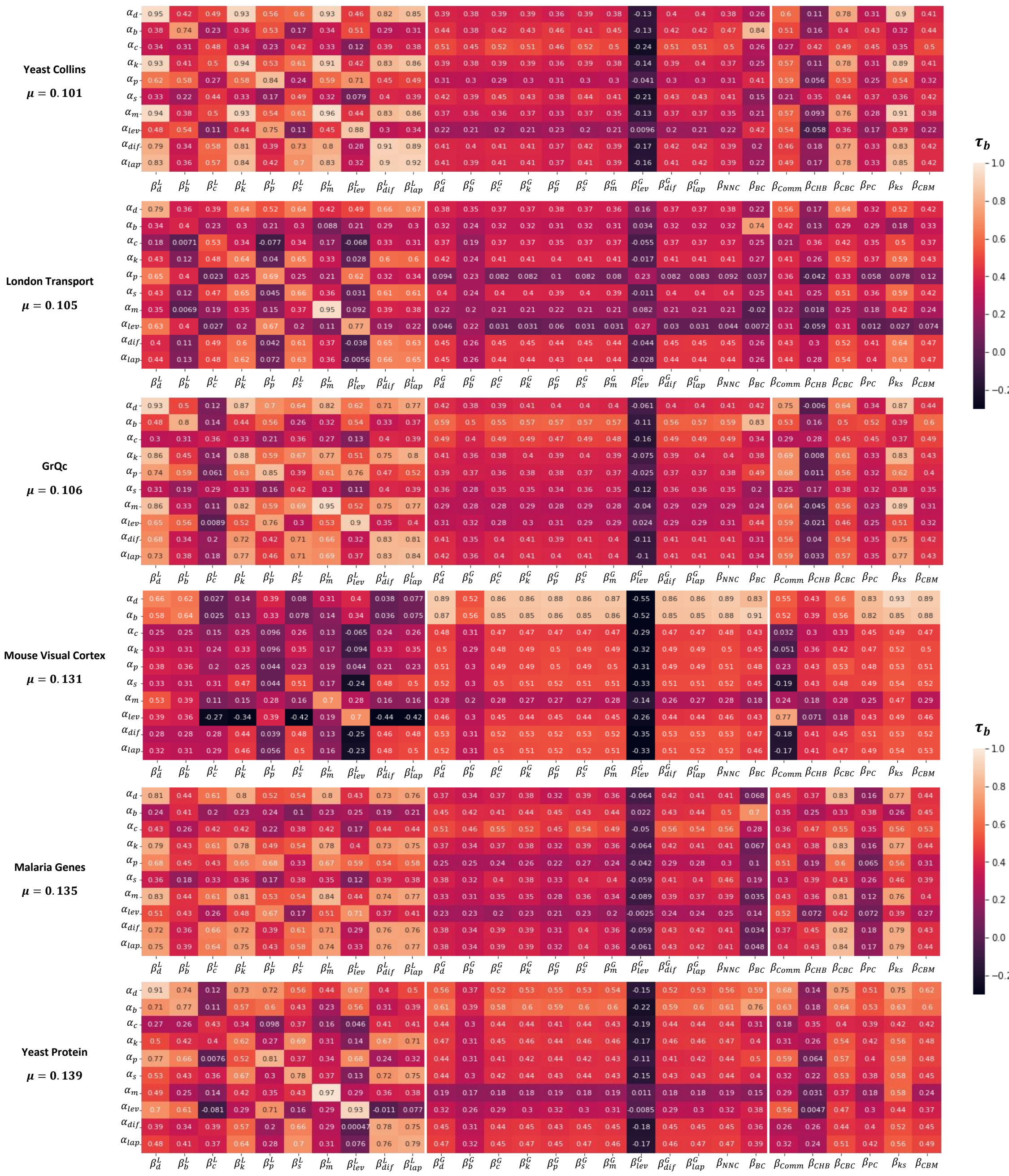

Figure 18. Heatmaps of Kendall's Tau correlation of the various combinations between classical (α_i) and community-aware (β_j) centrality measures in 6 real-world networks. Networks are sorted in ascending order according to their mixing parameter (μ). The mixing parameter is deduced after the community structure is uncovered using Louvain community detection algorithm. The classical centrality measures are: α_d = Degree, α_b = Betweenness, α_c = Closeness, α_k = Katz, α_p = PageRank, α_s = Subgraph, α_m = Maximum Neighborhood Component, α_{lev} = Leverage, α_{dif} = Diffusion, α_{lap} = Laplacian. The local community-aware centrality measures are: (β_d^L, β_b^L, β_c^L, β_k^L ,β_p^L, β_s^L, β_m^L, β_{lev}^L, β_{dif}^L, β_{lap}^L) = the local component of the classical centrality measures based on modular centrality. The global community-aware centrality measures are: (β_d^G, β_b^G, β_c^G, β_k^G ,β_p^G, β_s^G, β_m^G, β_{lev}^G, β_{dif}^G, β_{lap}^G) = the global component of the classical centrality measures based on modular centrality, β_{NNC} = Number of Neighboring Communities centrality, β_{BC} = Bridging centrality. The mixed community-aware centrality measures are: β_{Comm} = Comm centrality, β_{CHB} = Community Hub-Bridge centrality, β_{CBC} = Community-based centrality, β_{PC} = Participation Coefficient, β_{ks} = K-shell with Community centrality, β_{CBM} = Community-based Mediator centrality.

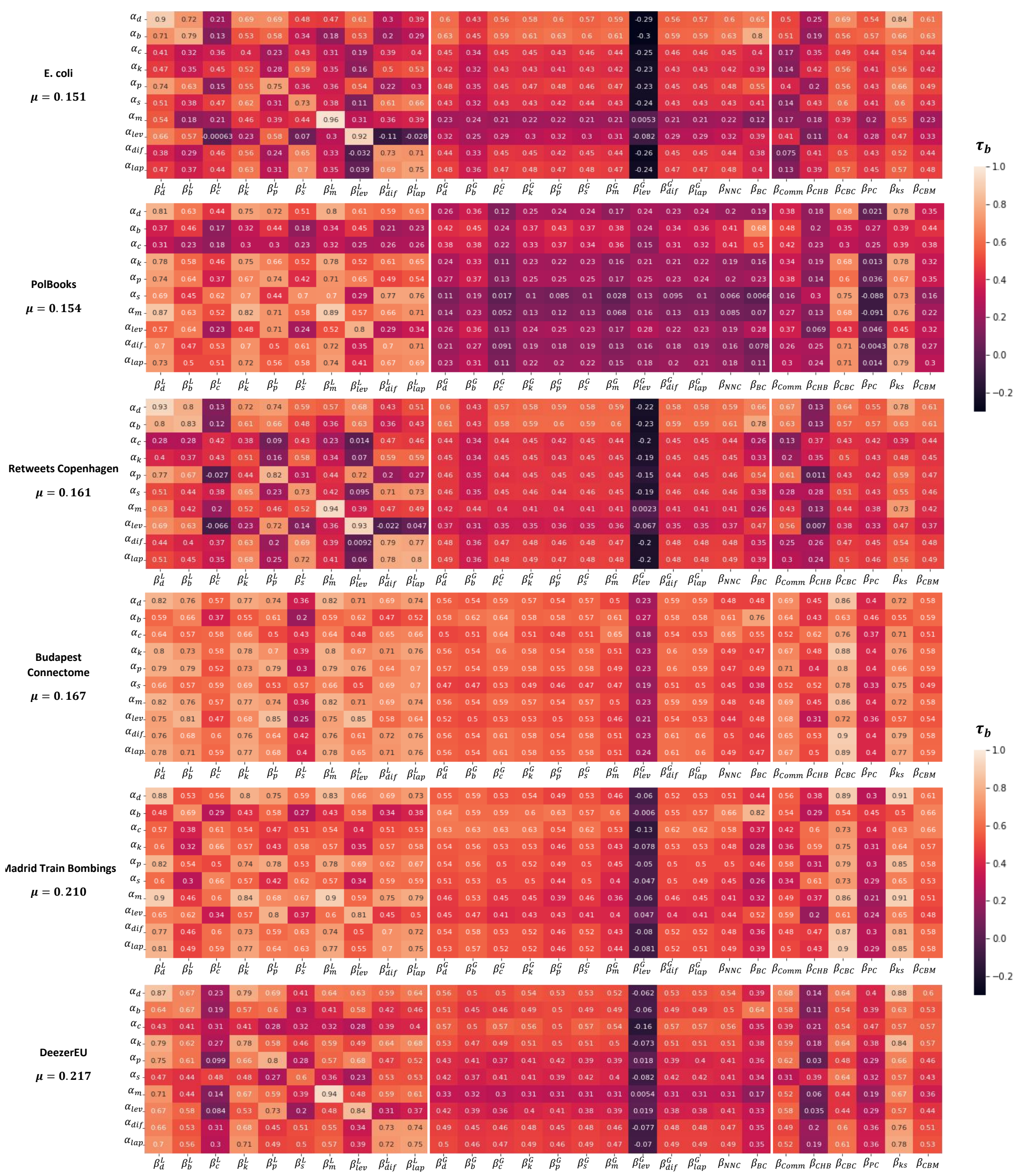

Figure 19. Heatmaps of Kendall's Tau correlation of the various combinations between classical (α_i) and community-aware (β_j) centrality measures in 6 real-world networks. Networks are sorted in ascending order according to their mixing parameter (μ). The mixing parameter is deduced after the community structure is uncovered using Louvain community detection algorithm. The classical centrality measures are: α_d = Degree, α_b = Betweenness, α_c = Closeness, α_k = Katz, α_p = PageRank, α_s = Subgraph, α_m = Maximum Neighborhood Component, α_{lev} = Leverage, α_{dif} = Diffusion, α_{lap} = Laplacian. The local community-aware centrality measures are: (β_d^L, β_b^L, β_c^L, β_k^L ,β_p^L, β_s^L, β_m^L, β_{lev}^L, β_{dif}^L, β_{lap}^L) = the local component of the classical centrality measures based on modular centrality. The global community-aware centrality measures are: (β_d^G, β_b^G, β_c^G, β_k^G ,β_p^G, β_s^G, β_m^G, β_{lev}^G, β_{dif}^G, β_{lap}^G) = the global component of the classical centrality measures based on modular centrality, β_{NNC} = Number of Neighboring Communities centrality, β_{BC} = Bridging centrality. The mixed community-aware centrality measures are: β_{Comm} = Comm centrality, β_{CHB} = Community Hub-Bridge centrality, β_{CBC} = Community-based centrality, β_{PC} = Participation Coefficient, β_{ks} = K-shell with Community centrality, β_{CBM} = Community-based Mediator centrality.

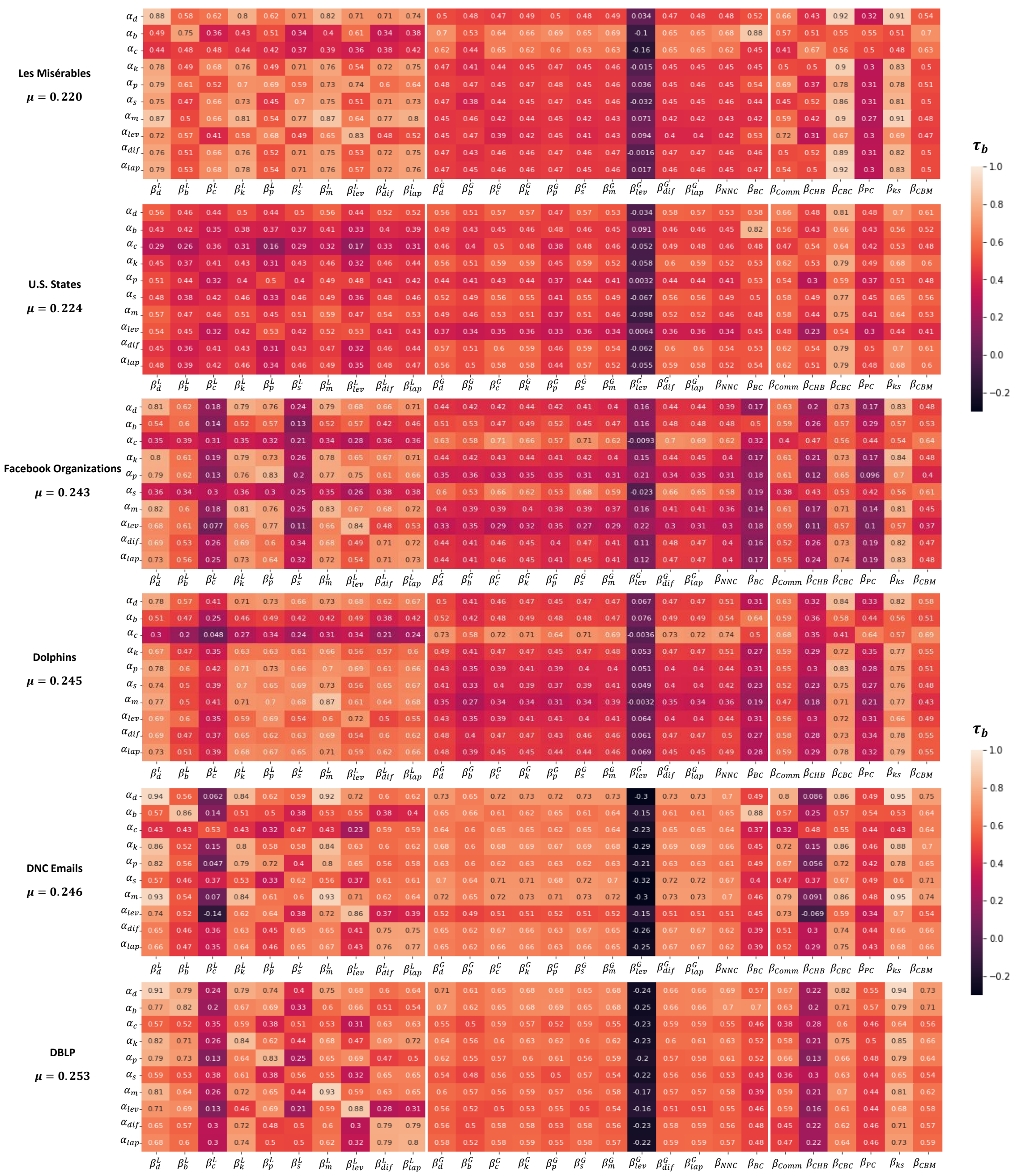

Figure 20. Heatmaps of Kendall's Tau correlation of the various combinations between classical (α_i) and community-aware (β_j) centrality measures in 6 real-world networks. Networks are sorted in ascending order according to their mixing parameter (μ). The mixing parameter is deduced after the community structure is uncovered using Louvain community detection algorithm. The classical centrality measures are: α_d = Degree, α_b = Betweenness, α_c = Closeness, α_k = Katz, α_p = PageRank, α_s = Subgraph, α_m = Maximum Neighborhood Component, α_{lev} = Leverage, α_{dif} = Diffusion, α_{lap} = Laplacian. The local community-aware centrality measures are: (β_d^L, β_b^L, β_c^L, β_k^L ,β_p^L, β_s^L, β_m^L, β_{lev}^L, β_{dif}^L, β_{lap}^L) = the local component of the classical centrality measures based on modular centrality. The global community-aware centrality measures are: (β_d^G, β_b^G, β_c^G, β_k^G ,β_p^G, β_s^G, β_m^G, β_{lev}^G, β_{dif}^G, β_{lap}^G) = the global component of the classical centrality measures based on modular centrality, β_{NNC} = Number of Neighboring Communities centrality, β_{BC} = Bridging centrality. The mixed community-aware centrality measures are: β_{Comm} = Comm centrality, β_{CHB} = Community Hub-Bridge centrality, β_{CBC} = Community-based centrality, β_{PC} = Participation Coefficient, β_{ks} = K-shell with Community centrality, β_{CBM} = Community-based Mediator centrality.

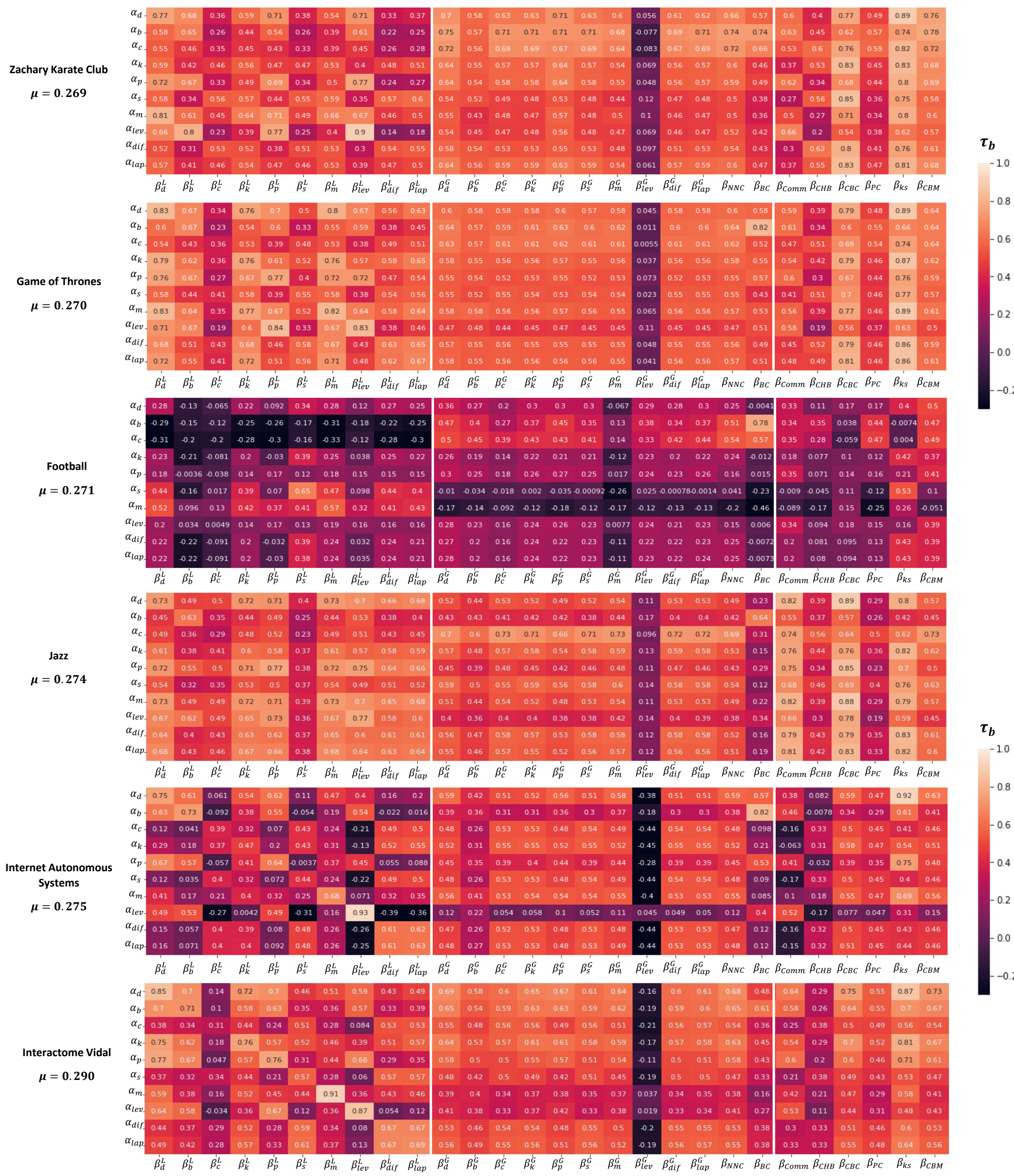

Figure 21. Heatmaps of Kendall's Tau correlation of the various combinations between classical (α_i) and community-aware (β_j) centrality measures in 6 real-world networks. Networks are sorted in ascending order according to their mixing parameter (μ). The mixing parameter is deduced after the community structure is uncovered using Louvain community detection algorithm. The classical centrality measures are: α_d = Degree, α_b = Betweenness, α_c = Closeness, α_k = Katz, α_p = PageRank, α_s = Subgraph, α_m = Maximum Neighborhood Component, α_{lev} = Leverage, α_{dif} = Diffusion, α_{lap} = Laplacian. The local community-aware centrality measures are: (β_d^L, β_b^L, β_c^L, β_k^L ,β_p^L, β_s^L, β_m^L, β_{lev}^L, β_{dif}^L, β_{lap}^L) = the local component of the classical centrality measures based on modular centrality. The global community-aware centrality measures are: (β_d^G, β_b^G, β_c^G, β_k^G ,β_p^G, β_s^G, β_m^G, β_{lev}^G, β_{dif}^G, β_{lap}^G) = the global component of the classical centrality measures based on modular centrality, β_{NNC} = Number of Neighboring Communities centrality, β_{BC} = Bridging centrality. The mixed community-aware centrality measures are: β_{Comm} = Comm centrality, β_{CHB} = Community Hub-Bridge centrality, β_{CBC} = Community-based centrality, β_{PC} = Participation Coefficient, β_{ks} = K-shell with Community centrality, β_{CBM} = Community-based Mediator centrality.

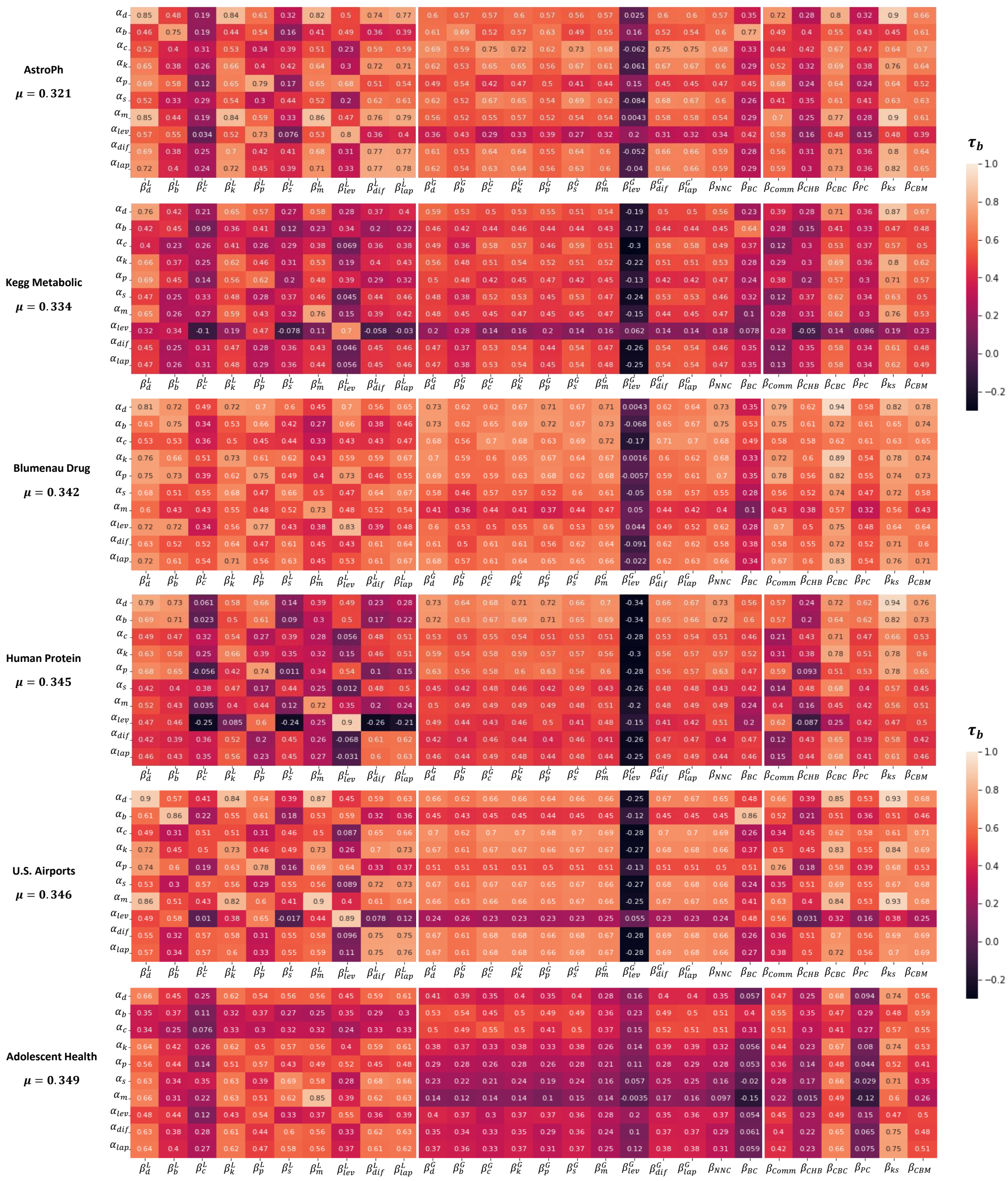

Figure 22. Heatmaps of Kendall's Tau correlation of the various combinations between classical (α_i) and community-aware (β_j) centrality measures in 6 real-world networks. Networks are sorted in ascending order according to their mixing parameter (μ). The mixing parameter is deduced after the community structure is uncovered using Louvain community detection algorithm. The classical centrality measures are: α_d = Degree, α_b = Betweenness, α_c = Closeness, α_k = Katz, α_p = PageRank, α_s = Subgraph, α_m = Maximum Neighborhood Component, α_{lev} = Leverage, α_{dif} = Diffusion, α_{lap} = Laplacian. The local community-aware centrality measures are: (β_d^L, β_b^L, β_c^L, β_k^L ,β_p^L, β_s^L, β_m^L, β_{lev}^L, β_{dif}^L, β_{lap}^L) = the local component of the classical centrality measures based on modular centrality. The global community-aware centrality measures are: (β_d^G, β_b^G, β_c^G, β_k^G ,β_p^G, β_s^G, β_m^G, β_{lev}^G, β_{dif}^G, β_{lap}^G) = the global component of the classical centrality measures based on modular centrality, β_{NNC} = Number of Neighboring Communities centrality, β_{BC} = Bridging centrality. The mixed community-aware centrality measures are: β_{Comm} = Comm centrality, β_{CHB} = Community Hub-Bridge centrality, β_{CBC} = Community-based centrality, β_{PC} = Participation Coefficient, β_{ks} = K-shell with Community centrality, β_{CBM} = Community-based Mediator centrality.

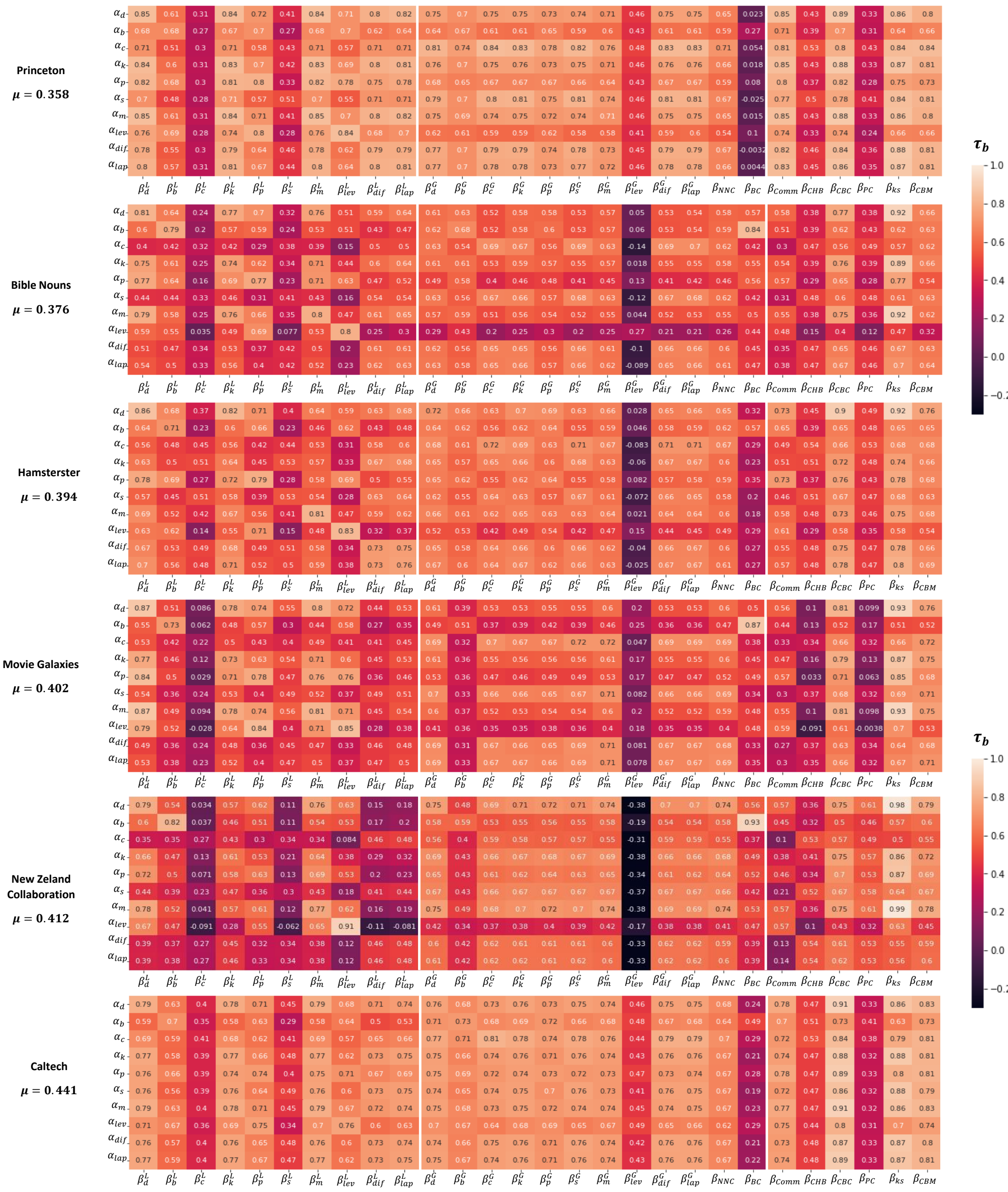

Figure 23. Heatmaps of Kendall's Tau correlation of the various combinations between classical (α_i) and community-aware (β_j) centrality measures in 6 real-world networks. Networks are sorted in ascending order according to their mixing parameter (μ). The mixing parameter is deduced after the community structure is uncovered using Louvain community detection algorithm. The classical centrality measures are: α_d = Degree, α_b = Betweenness, α_c = Closeness, α_k = Katz, α_p = PageRank, α_s = Subgraph, α_m = Maximum Neighborhood Component, α_{lev} = Leverage, α_{dif} = Diffusion, α_{lap} = Laplacian. The local community-aware centrality measures are: (β_d^L, β_b^L, β_c^L, β_k^L ,β_p^L, β_s^L, β_m^L, β_{lev}^L, β_{dif}^L, β_{lap}^L) = the local component of the classical centrality measures based on modular centrality. The global community-aware centrality measures are: (β_d^G, β_b^G, β_c^G, β_k^G ,β_p^G, β_s^G, β_m^G, β_{lev}^G, β_{dif}^G, β_{lap}^G) = the global component of the classical centrality measures based on modular centrality, β_{NNC} = Number of Neighboring Communities centrality, β_{BC} = Bridging centrality. The mixed community-aware centrality measures are: β_{Comm} = Comm centrality, β_{CHB} = Community Hub-Bridge centrality, β_{CBC} = Community-based centrality, β_{PC} = Participation Coefficient, β_{ks} = K-shell with Community centrality, β_{CBM} = Community-based Mediator centrality.

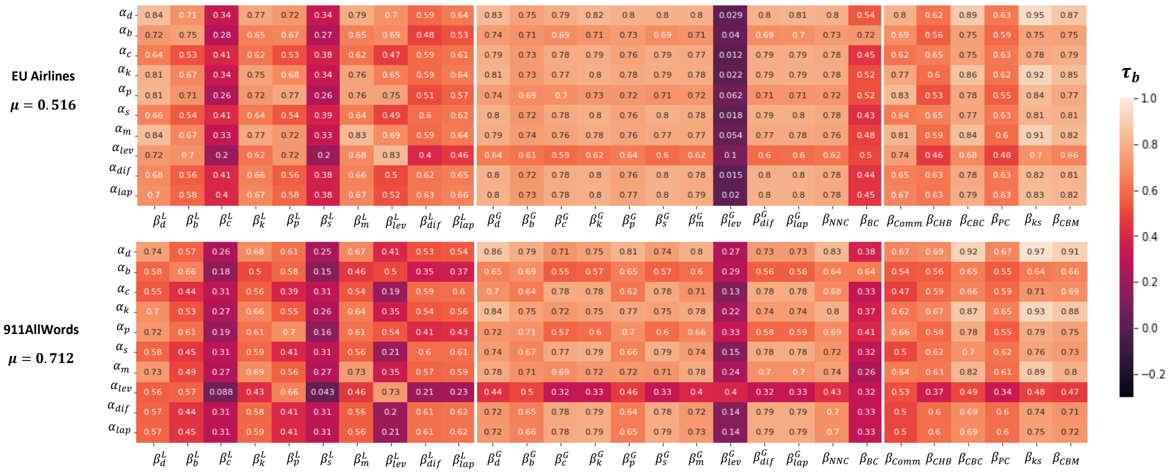

Figure 24. Heatmaps of Kendall's Tau correlation of the various combinations between classical (α_i) and community-aware (β_j) centrality measures in 6 real-world networks. Networks are sorted in ascending order according to their mixing parameter (μ). The mixing parameter is deduced after the community structure is uncovered using Louvain community detection algorithm. The classical centrality measures are: α_d = Degree, α_b = Betweenness, α_c = Closeness, α_k = Katz, α_p = PageRank, α_s = Subgraph, α_m = Maximum Neighborhood Component, α_{lev} = Leverage, α_{dif} = Diffusion, α_{lap} = Laplacian. The local community-aware centrality measures are: (β_d^L, β_b^L, β_c^L, β_k^L ,β_p^L, β_s^L, β_m^L, β_{lev}^L, β_{dif}^L, β_{lap}^L) = the local component of the classical centrality measures based on modular centrality. The global community-aware centrality measures are: (β_d^G, β_b^G, β_c^G, β_k^G ,β_p^G, β_s^G, β_m^G, β_{lev}^G, β_{dif}^G, β_{lap}^G) = the global component of the classical centrality measures based on modular centrality, β_{NNC} = Number of Neighboring Communities centrality, β_{BC} = Bridging centrality. The mixed community-aware centrality measures are: β_{Comm} = Comm centrality, β_{CHB} = Community Hub-Bridge centrality, β_{CBC} = Community-based centrality, β_{PC} = Participation Coefficient, β_{ks} = K-shell with Community centrality, β_{CBM} = Community-based Mediator centrality.

Table 1. Macroscopic topological properties of real-world networks. N is the total number of nodes. $|E|$ is the number of edges. $< k >$ is the average degree. $< d >$ is the average distance. ν is the density. ζ is the transitivity. $k_{nn}(k)$ is the assortativity. D is the diameter. ε is the efficiency. γ_{pred} is the estimated exponent of the degree distribution. * indicates the largest connected component if the network is disconnected.

Network	N	E	$< k >$	$< d >$	ν	ζ	$k_{nn}(k)$	D	ε	γ_{pred}
Zachary Karate Club	34	78	4.58	2.40	0.139	0.255	-0.475	5	0.492	2.094
U.S. States	49	107	4.36	4.16	0.090	0.406	0.233	11	0.388	1.478
Dolphins	62	159	5.12	3.35	0.084	0.308	-0.043	8	0.379	1.470
Madrid Train Bombings	64	243	7.59	2.69	0.120	0.561	0.029	6	0.448	1.425
Blumenau Drug	75	181	4.83	2.81	0.065	0.108	-0.314	6	0.407	2.437
Les Misérables	77	254	6.59	2.64	0.086	0.498	-0.165	5	0.435	1.689
PolBooks	105	441	8.40	3.07	0.080	0.348	-0.127	7	0.397	2.600
Game of Thrones	107	352	6.58	2.90	0.062	0.328	-0.130	6	0.398	2.022
Movie Galaxies	109	326	5.98	2.06	0.055	0.162	-0.360	3	0.508	2.392
Football	115	613	10.66	2.50	0.093	0.407	0.162	4	0.450	1.327
Marvel Partnerships*	181	224	2.48	7.92	0.014	0.216	-0.207	21	0.166	1.724
Mouse Visual Cortex	193	214	2.21	4.27	0.015	0.004	-0.844	8	0.271	2.060
Internet Topology Cogentco	197	243	2.47	10.51	0.013	0.019	0.027	28	0.137	1.660
Jazz	198	2,742	27.69	2.23	0.140	0.520	0.020	6	0.513	1.477
Malaria Genes	307	2,812	18.32	3.52	0.060	0.587	0.599	8	0.349	2.443
E. coli Transcription*	329	456	2.77	4.84	0.008	0.023	-0.263	13	0.245	2.295
Facebook Friends*	329	1,954	11.88	3.58	0.036	0.512	0.074	9	0.326	2.015
London Transport	369	430	2.33	13.73	0.006	0.051	0.137	39	0.101	1.682
NetSci	379	914	4.83	6.04	0.012	0.430	-0.081	17	0.203	2.386
EU Airlines	417	2,953	14.16	2.76	0.034	0.304	-0.152	7	0.400	2.061
Reptiles*	496	984	3.96	7.93	0.008	0.419	0.345	21	0.155	2.120
U.S. Airports	500	2,980	11.92	2.99	0.023	0.351	-0.267	7	0.371	1.702
Retweets Copenhagen	761	1,029	2.70	5.35	0.003	0.060	-0.099	14	0.208	2.178
Caltech*	762	16,651	43.70	2.23	0.057	0.291	-0.066	6	0.461	3.005
DNC Emails*	849	10,384	24.46	2.76	0.029	0.548	-0.133	8	0.399	1.417
Board of Directors*	854	2,745	6.43	6.67	0.008	0.624	0.051	17	0.176	2.150
Yeast Collins*	1,004	8,319	16.57	5.55	0.016	0.617	0.556	15	0.220	2.120
Budapest Connectome	1,015	70,654	139.22	2.22	0.137	0.558	0.215	5	0.510	1.696
CS Ph.D.*	1,025	1,043	2.03	11.74	0.001	0.002	-0.253	28	0.105	1.928
EuroRoad*	1,039	1,305	2.51	18.39	0.002	0.035	0.090	62	0.077	1.660
Yeast Protein*	1,458	1,993	2.73	6.81	0.001	0.051	-0.207	19	0.163	2.185
New Zealand Collaboration*	1,463	4,246	5.80	2.75	0.003	0.063	-0.340	6	0.381	2.112
Bible Nouns*	1,707	9,059	10.61	3.38	0.006	0.162	-0.052	8	0.320	2.134
Hamsterster*	1,788	12,476	13.49	3.45	0.007	0.090	-0.088	14	0.317	1.868
Kegg Metabolic*	1,865	5,769	6.19	3.12	0.003	0.030	-0.224	8	0.342	2.331
Human Protein*	2,217	6,418	5.78	3.84	0.002	0.007	-0.331	10	0.281	2.240
Adolescent Health	2,539	10,455	8.23	4.55	0.003	0.141	0.251	10	0.234	1.876
Interactome Vidal*	2,783	6,007	4.32	4.84	0.002	0.035	-0.137	13	0.223	2.192
Ego Facebook	4,039	88,234	43.69	3.69	0.010	0.519	0.063	8	0.306	2.291
GrQc	4,158	13,422	6.45	6.04	0.001	0.628	0.639	17	0.179	2.042
U.S. Power Grid	4,941	6,594	2.66	18.98	0.005	0.103	0.003	46	0.062	1.669
Facebook Organizations	5,524	94,219	34.11	3.50	0.006	0.222	0.103	9	0.313	2.279
Facebook Politician Pages	5,908	41,729	14.12	4.66	0.002	0.301	0.018	14	0.231	2.664
Internet Autonomous Systems	6,474	12,572	3.88	3.70	0.0006	0.009	-0.181	9	0.290	2.356
Princeton*	6,575	293,307	89.21	2.67	0.013	0.163	0.090	9	0.400	3.046
PGP	10,680	24,316	4.55	7.48	0.0004	0.378	0.238	24	0.147	2.804
DBLP*	12,494	49,579	7.94	4.42	0.0006	0.062	-0.046	10	0.240	2.324
911AllWords	13,308	148,035	22.25	3.12	0.0016	0.106	-0.108	10	0.334	2.023
AstroPh*	17,903	196,972	22.00	4.19	0.001	0.317	0.201	14	0.255	2.891
DeezerEU	28,281	92,752	6.55	6.44	0.002	0.095	0.104	21	0.169	2.273

Table 2. Mesoscopic topological properties of real-world networks with community structure unveiled using Infomap. μ is the mixing parameter. Q is the modularity. χ is the internal degree. φ is the internal distance. ψ is the internal density. η is the Max-ODF. ι is the Average-ODF. κ is the Flake-ODF. $\backepsilon$ is the embeddedness. $\hbar$ is the hub dominance.

Network	μ	Q	χ	φ	ψ	η	ι	κ	$\backepsilon$	$\hbar$
EU Airlines	0.073	0.109	3.97	1.49	0.596	0.559	0.266	0.388	0.734	0.874
Ego Facebook	0.077	0.814	14.69	1.53	0.538	0.457	0.244	0.176	0.755	0.872
U.S. Airports	0.084	0.161	2.30	1.37	0.701	0.508	0.347	0.530	0.652	0.918
Budapest Connectome	0.088	0.525	90.75	1.22	0.295	0.412	0.070	0.009	0.680	0.921
Facebook Politician Pages	0.111	0.836	5.33	1.85	0.402	0.466	0.180	0.199	0.819	0.756
Facebook Friends	0.112	0.688	5.17	1.54	0.554	0.486	0.231	0.286	0.769	0.838
Madrid Train Bombings	0.115	0.309	4.25	1.59	0.526	0.440	0.280	0.328	0.719	0.865
Yeast Collins	0.122	0.747	6.99	1.52	0.618	0.431	0.136	0.129	0.864	0.868
Malaria Genes	0.128	0.625	10.42	1.47	0.601	0.538	0.188	0.146	0.812	0.825
CS Ph.D.	0.128	0.859	1.70	1.97	0.306	0.307	0.068	0.068	0.931	0.793
Reptiles	0.145	0.811	2.77	1.85	0.443	0.400	0.133	0.127	0.866	0.722
PolBooks	0.145	0.522	5.39	1.57	0.511	0.516	0.245	0.177	0.754	0.769
NetSci	0.147	0.810	3.69	1.53	0.537	0.320	0.114	0.083	0.885	0.914
Marvel Partnerships	0.147	0.808	2.01	1.86	0.408	0.423	0.121	0.141	0.879	0.755
911AllWords	0.153	0.052	1.72	1.63	0.603	0.699	0.548	0.834	0.452	0.820
Mouse Visual Cortex	0.154	0.740	1.84	1.84	0.161	0.345	0.090	0.144	0.909	1
U.S. Power Grid	0.166	0.830	2.08	2.56	0.242	0.435	0.130	0.140	0.869	0.475
Board of Directors	0.167	0.817	5.16	1.49	0.578	0.459	0.141	0.118	0.859	0.904
PGP	0.172	0.813	2.40	1.97	0.388	0.381	0.097	0.102	0.902	0.727
Zachary Karate Club	0.179	0.402	3.47	1.66	0.404	0.294	0.163	0.027	0.836	0.844
London Transport	0.198	0.779	1.83	2.49	0.313	0.497	0.170	0.273	0.830	0.452
GrQc	0.200	0.779	3.73	1.87	0.394	0.461	0.155	0.151	0.844	0.765
EuroRoad	0.202	0.786	1.94	2.70	0.254	0.463	0.161	0.205	0.838	0.398
Internet Topology Cogentco	0.206	0.751	1.91	2.23	0.326	0.487	0.179	0.199	0.821	0.473
DNC Emails	0.211	0.416	5.70	1.40	0.633	0.561	0.323	0.466	0.677	0.925
Dolphins	0.213	0.525	3.41	1.75	0.467	0.532	0.292	0.312	0.707	0.695
U.S. States	0.224	0.596	3.35	1.64	0.479	0.475	0.180	0.144	0.819	0.767
Yeast Protein	0.240	0.749	1.94	2.04	0.324	0.432	0.149	0.173	0.850	0.728
E. coli Transcription	0.250	0.687	1.69	1.66	0.466	0.464	0.208	0.315	0.791	0.883
Game of Thrones	0.267	0.482	3.80	1.63	0.421	0.503	0.214	0.207	0.786	0.937
Jazz	0.267	0.443	15.51	1.51	0.540	0.612	0.280	0.268	0.719	0.835
Les Misérables	0.267	0.551	4.30	1.45	0.584	0.441	0.178	0.141	0.821	0.938
Retweets Copenhagen	0.287	0.695	1.80	1.93	0.338	0.433	0.129	0.160	0.870	0.778
Football	0.293	0.604	7.58	1.27	0.751	0.435	0.293	0.056	0.293	0.821
Hamsterster	0.298	0.391	3.52	1.86	0.369	0.623	0.317	0.417	0.682	0.746
Blumenau Drug	0.337	0.441	2.58	1.845	0.326	0.571	0.246	0.279	0.754	0.705
Human Protein	0.360	0.466	2.03	2.06	0.253	0.595	0.281	0.399	0.718	0.786
Princeton	0.365	0.417	15.74	1.77	0.480	0.708	0.459	0.584	0.540	0.725
Facebook Organizations	0.366	0.585	15.09	1.95	0.267	0.716	0.461	0.517	0.539	0.633
Interactome Vidal	0.405	0.576	2.08	2.20	0.297	0.559	0.252	0.344	0.748	0.648
Caltech	0.410	0.389	13.35	1.79	0.427	0.657	0.425	0.488	0.574	0.731
AstroPh	0.420	0.563	6.62	1.89	0.410	0.627	0.302	0.324	0.697	0.740
Internet Autonomous Systems	0.428	0.547	1.78	1.94	0.350	0.508	0.312	0.480	0.687	0.810
DeezerEU	0.429	0.565	2.59	2.48	0.223	0.573	0.321	0.423	0.678	0.570
Movie Galaxies	0.429	0.401	2.89	1.57	0.498	0.547	0.379	0.382	0.621	0.919
Adolescent Health	0.431	0.566	3.47	2.29	0.284	0.704	0.473	0.582	0.526	0.511
DBLP	0.433	0.546	2.54	2.26	0.195	0.583	0.257	0.358	0.743	0.696
Kegg Metabolic	0.466	0.437	1.77	1.79	0.529	0.592	0.427	0.697	0.573	0.736
Bible Nouns	0.496	0.460	4.00	1.88	0.358	0.663	0.343	0.416	0.657	0.757
New Zealand Collaboration	0.564	0.401	2.28	1.73	0.386	0.645	0.384	0.552	0.616	0.891

Table 3. Simple linear regression estimates using ordinary least squares. Independent variables are the macroscopic and mesoscopic properties considered individually. Dependent variables are the mean of the correlation between classical and local/global community-aware centrality measures denoted as Mean_L and Mean_G, respectively. ω is the estimated coefficient. ε is the standard error. The confidence interval is based on 95% likelihood. R^2 is the coefficient of determination. * indicates $p \leq 0.05$. A bold font and * indicate $p \leq 0.01$.

Dependent Variable	Independent Variable	ω	ε	T	$p > \|T\|$	Confidence Interval	R^2
Mean_L	**Density***	1.268	0.420	3.019	0.004	[0.423; 2.112]	0.160
	**Transitivity***	0.357	0.077	4.656	0.00003	[0.203; 0.511]	0.311
	Assortativity	0.136	0.069	1.979	0.054	[-0.002; 0.275]	0.075
	Average distance*	-0.010	0.005	-2.024	0.049	[-0.020 -0.00006]	0.079
	Diameter	-0.003	0.002	-1.753	0.086	[-0.006; 0.0001]	0.060
	**Efficiency***	0.388	0.145	2.675	0.010	[0.096; 0.680]	0.130
	Degree distribution exponent	-0.002	0.045	-0.042	0.966	[-0.091; 0.088]	0.00004
	Mixing parameter*	-0.294	0.141	-2.090	0.042	[-0.577; -0.011]	0.083
	Modularity	-0.174	0.108	-1.607	0.115	[-0.391; 0.044]	0.051
	Internal distance*	-0.121	0.055	-2.206	0.032	[-0.230; -0.011]	0.092
	Internal density	0.244	0.134	1.819	0.075	[-0.026; 0.513]	0.064
	Max-ODF	0.153	0.179	0.850	0.400	[-0.208; 0.513]	0.015
	Average-ODF	0.038	0.165	0.231	0.819	[-0.294; 0.370]	0.001
	Flake-ODF	-0.037	0.103	-0.358	0.722	[-0.245; 0.171]	0.003
	Embeddedness	-0.118	0.168	-0.700	0.487	[-0.456; 0.221]	0.010
	Hub dominance	0.151	0.137	1.100	0.277	[-0.125; 0.426]	0.025
Mean_G	Density	-0.359	0.369	-0.973	0.336	[-1.102; 0.383]	0.019
	**Transitivity***	-0.205	0.069	-2.966	0.005	[-0.344; -0.066]	0.155
	Assortativity*	-0.140	0.055	-2.564	0.014	[-0.250; -0.030]	0.120
	Average distance	-0.006	0.004	-1.381	0.174	[-0.014; 0.003]	0.038
	Diameter	-0.002	0.001	-1.097	0.278	[-0.004; 0.001]	0.024
	Efficiency	0.131	0.125	1.048	0.300	[-0.120; 0.383]	0.022
	Degree distribution exponent	0.061	0.037	1.661	0.103	[-0.013; 0.134]	0.055
	**Mixing parameter***	0.572	0.087	6.608	2.94e-08	[0.398; 0.747]	0.476
	Modularity	-0.135	0.076	-1.759	0.085	[-0.288; 0.019]	0.061
	Internal distance	0.073	0.045	1.610	0.114	[-0.018; 0.165]	0.051
	**Internal density***	-0.295	0.104	-2.824	0.007	[-0.504; -0.085]	0.142
	**Max-ODF***	0.402	0.135	2.976	0.005	[0.130; 0.674]	0.156
	Average-ODF*	0.262	0.129	2.032	0.048	[0.003; 0.522]	0.079
	Flake-ODF*	0.199	0.079	2.508	0.016	[0.039; 0.358]	0.116
	Embeddedness	-0.243	0.133	-1.826	0.074	[-0.511; 0.025]	0.065
	Hub dominance	0.007	0.113	0.063	0.950	[-0.220; 0.234]	0.00008

Table 4. Simple linear regression estimates using weighted least squares. Independent variables are the macroscopic and mesoscopic properties considered individually. Dependent variables are the mean of the correlation between classical and local/global community-aware centrality measures denoted as Mean_L and Mean_G, respectively. ω is the estimated coefficient. ε is the standard error. The confidence interval is based on 95% likelihood. R^2 is the coefficient of determination. * indicates $p \leq 0.05$. A bold font and * indicate $p \leq 0.01$.

Dependent Variable	Independent Variable	ω	ε	T	$p > \|T\|$	Confidence Interval	R^2
Mean_L	**Density***	1.260	0.324	3.894	0.0003	[0.609; 1.911]	0.240
	**Transitivity***	0.364	0.077	4.716	0.00002	[0.209; 0.519]	0.317
	Assortativity	0.074	0.056	1.318	0.194	[-0.039; 0.187]	0.035
	Average distance*	-0.009	0.004	-2.113	0.040	[-0.018; -0.0004]	0.085
	Diameter*	-0.003	0.001	-2.217	0.031	[-0.005; -0.0002]	0.093
	**Efficiency***	0.372	0.141	2.632	0.010	[0.088; 0.656]	0.126
	Degree distribution exponent	0.032	0.0425	0.754	0.454	[-0.053; 0.117]	0.012
	Mixing parameter*	-0.494	0.196	-2.523	0.015	[-0.887; -0.100]	0.117
	Modularity*	-0.212	0.090	-2.357	0.023	[-0.392; -0.031]	0.104
	Internal distance*	-0.121	0.059	-2.039	0.047	[-0.239; -0.002]	0.080
	Internal density	-0.008	0.147	-0.057	0.954	[-0.304; 0.287]	0.00007
	Max-ODF	0.155	0.173	0.898	0.374	[-0.193; 0.503]	0.017
	Average-ODF	0.042	0.168	0.248	0.805	[-0.296; 0.379]	0.001
	Flake-ODF	-0.027	0.114	-0.233	0.817	[-0.256; 0.203]	0.001
	Embeddedness	-0.116	0.168	-0.689	0.494	[-0.453; 0.222]	0.010
	Hub dominance*	0.223	0.110	2.031	0.048	[0.002; 0.444]	0.080
Mean_G	Density	-0.369	0.449	-0.821	0.416	[-1.272; 0.534]	0.014
	**Transitivity***	-0.200	0.070	-2.846	0.006	[-0.339; -0.058]	0.144
	Assortativity*	-0.141	0.054	-2.595	0.013	[-0.250; -0.032]	0.123
	Average distance*	-0.003	0.001	-2.68	0.011	[-0.005; -0.001]	0.130
	Diameter	-0.0008	0.0004	-1.889	0.065	[-0.002; 0.00005]	0.069
	Efficiency	0.182	0.111	1.644	0.107	[-0.041; 0.405]	0.053
	Degree distribution exponent	0.043	0.038	1.135	0.262	[-0.033; 0.119]	0.027
	**Mixing parameter***	0.546	0.084	6.519	4.02e-08	[0.377; 0.714]	0.470
	**Modularity***	-0.264	0.079	-3.357	0.002	[-0.422; -0.106]	0.190
	Internal distance	0.055	0.042	1.286	0.205	[-0.031; 0.140]	0.033
	Internal density*	-0.268	0.103	-2.604	0.012	[-0.475; -0.061]	0.124
	Max-ODF*	0.333	0.136	2.449	0.018	[0.060; 0.607]	0.111
	Average-ODF	0.250	0.147	1.703	0.095	[-0.045; 0.545]	0.057
	**Flake-ODF***	0.277	0.094	2.951	0.005	[0.088; 0.465]	0.154
	Embeddedness	-0.213	0.151	-1.413	0.164	[-0.516; 0.090]	0.040
	Hub dominance	0.055	0.089	0.615	0.541	[-0.124; 0.233]	0.008

References

1. Jalili, M. *et al.* Centiserver: a comprehensive resource, web-based application and r package for centrality analysis. *PloS one* **10**, e0143111 (2015).
2. Ghalmane, Z., El Hassouni, M., Cherifi, C. & Cherifi, H. Centrality in modular networks. *EPJ Data Sci.* **8**, 15 (2019).
3. Ghalmane, Z., El Hassouni, M. & Cherifi, H. Immunization of networks with non-overlapping community structure. *Soc. Netw. Analysis Min.* **9**, 45 (2019).
4. Hwang, W., Cho, Y.-r., Zhang, A. & Ramanathan, M. Bridging centrality: identifying bridging nodes in scale-free networks. In *Proceedings of the 12th ACM SIGKDD international conference on Knowledge discovery and data mining*, 20–23 (2006).
5. Gupta, N., Singh, A. & Cherifi, H. Centrality measures for networks with community structure. *Phys. A: Stat. Mech. its Appl.* **452**, 46–59 (2016).
6. Tulu, M. M., Hou, R. & Younas, T. Identifying influential nodes based on community structure to speed up the dissemination of information in complex network. *IEEE Access* **6**, 7390–7401 (2018).
7. Zhao, Z., Wang, X., Zhang, W. & Zhu, Z. A community-based approach to identifying influential spreaders. *Entropy* **17**, 2228–2252 (2015).
8. Guimera, R. & Amaral, L. A. N. Functional cartography of complex metabolic networks. *nature* **433**, 895–900 (2005).
9. Luo, S.-L., Gong, K. & Kang, L. Identifying influential spreaders of epidemics on community networks. *arXiv preprint arXiv:1601.07700* (2016).
10. Barabási, A.-L. *et al. Network science* (Cambridge university press, 2016).
11. Latora, V. & Marchiori, M. Efficient behavior of small-world networks. *Phys. review letters* **87**, 198701 (2001).
12. Newman, M. E. The structure and function of complex networks. *SIAM review* **45**, 167–256 (2003).
13. Jebabli, M., Cherifi, H., Cherifi, C. & Hamouda, A. Community detection algorithm evaluation with ground-truth data. *Phys. A: Stat. Mech. its Appl.* **492**, 651–706 (2018).
14. Yang, J. & Leskovec, J. Defining and evaluating network communities based on ground-truth. *Knowl. Inf. Syst.* **42**, 181–213 (2015).
15. Orman, G. K., Labatut, V. & Cherifi, H. Comparative evaluation of community detection algorithms: a topological approach. *J. Stat. Mech. Theory Exp.* **2012**, P08001 (2012).
16. Wooldridge, J. M. *Introductory econometrics: A modern approach* (Nelson Education, 2016).
17. Rosvall, M. & Bergstrom, C. T. Maps of random walks on complex networks reveal community structure. *Proc. Natl. Acad. Sci.* **105**, 1118–1123 (2008).
18. Blondel, V. D., Guillaume, J.-L., Lambiotte, R. & Lefebvre, E. Fast unfolding of communities in large networks. *J. statistical mechanics: theory experiment* **2008**, P10008 (2008).
19. Lusseau, D. *et al.* The bottlenose dolphin community of doubtful sound features a large proportion of long-lasting associations. *Behav. Ecol. Sociobiol.* **54**, 396–405 (2003).
20. Rossi, R. A. & Ahmed, N. K. The network data repository with interactive graph analytics and visualization. In *AAAI* (2015). [Online]. Available: http://networkrepository.com.
21. Clauset, A., Tucker, E. & Sainz, M. The colorado index of complex networks (2016). [Online]. Available: https://icon.colorado.edu/.
22. Latora, V., Nicosia, V. & Russo, G. *Complex networks: principles, methods and applications* (Cambridge University Press, 2017). [Online]. Available: https://www.complex-networks.net/datasets.html.
23. Rozemberczki, B. & Sarkar, R. Characteristic functions on graphs: Birds of a feather, from statistical descriptors to parametric models (2020). 2005.07959.
24. Peixoto, T. P. The netzschleuder network catalogue and repository (2020). [Online]. Available: https://networks.skewed.de/.
25. Kunegis, J. Handbook of network analysis [konect–the koblenz network collection]. *arXiv preprint arXiv:1402.5500* (2014).
